# Low-dimensional controllability of brain networks

**DOI:** 10.1371/journal.pcbi.1012691

**Published:** 2025-01-07

**Authors:** Remy Ben Messaoud, Vincent Le Du, Camile Bousfiha, Marie-Constance Corsi, Juliana Gonzalez-Astudillo, Brigitte Charlotte Kaufmann, Tristan Venot, Baptiste Couvy-Duchesne, Raffaella Migliaccio, Charlotte Rosso, Paolo Bartolomeo, Mario Chavez, Fabrizio De Vico Fallani

**Affiliations:** 1 Inria Paris, Paris, France; 2 Sorbonne Université, Paris Brain Institute, CNRS, Inserm, AP-HP, Hôpital de la Pitié-Salpêtrière, Paris, France; 3 Institute for Molecular Bioscience, University of Queensland, St Lucia, Australia; 4 Department of Neurology, Institute of Memory and Alzheimer’s Disease, Centre of Excellence of Neurodegenerative Disease, Hôpital Pitié-Salpêtrière, Paris, France; 5 Urgences Cérébro-Vasculaires, DMU Neurosciences, Hôpital Pitié-Salpêtrière, Paris, France; University of Chieti-Pescara, ITALY

## Abstract

Identifying the driver nodes of a network has crucial implications in biological systems from unveiling causal interactions to informing effective intervention strategies. Despite recent advances in network control theory, results remain inaccurate as the number of drivers becomes too small compared to the network size, thus limiting the concrete usability in many real-life applications. To overcome this issue, we introduced a framework that integrates principles from spectral graph theory and output controllability to project the network state into a smaller topological space formed by the Laplacian network structure. Through extensive simulations on synthetic and real networks, we showed that a relatively low number of projected components can significantly improve the control accuracy. By introducing a new low-dimensional controllability metric we experimentally validated our method on N = 6134 human connectomes obtained from the UK-biobank cohort. Results revealed previously unappreciated influential brain regions, enabled to draw directed maps between differently specialized cerebral systems, and yielded new insights into hemispheric lateralization. Taken together, our results offered a theoretically grounded solution to deal with network controllability and provided insights into the causal interactions of the human brain.

## Author summary

Identifying control nodes in complex networks is essential for understanding and influencing biological systems. However, existing network control methods often fall short when the number of driver nodes is small relative to the network size, limiting their practical application. To address this, we developed a novel framework combining spectral graph theory and output controllability, enabling us to reduce the system into a smaller, manageable space using network Laplacian projections. Extensive testing on synthetic and real-world networks showed that a limited number of projected components greatly improved control accuracy. We applied our method to 6,134 human brain connectomes from the UK Biobank dataset, revealing influential brain regions and mapping directed interactions across cerebral systems. Our findings offer new insights into brain organization and hemispheric lateralization, providing a robust solution for network controllability in biological systems.

## Introduction

The ability to influence the behavior of a complex system, beyond its mere description, leads to a more profound comprehension of it. Determining where and how to intervene to favor desired dynamics can reveal the driver nodes in interconnected systems such as gene regulatory and brain networks as well as inform intervention strategies to counteract diseases [1].

In particular, brain controllability has both fundamental and practical implications in neuroscience from studying the endogenous processes that underlie cognitive control to modeling the effects of exogenous stimuli on behavior such as open/closed-loop neuromodulation [2].

In recent years, network control theory has provided an unprecedented rigorous mathematical framework to study brain controllability from a theoretical perspective [3]. By modeling how information is stipulated to pass along the underlying network structure, controllability informs on the dynamical properties of the brain areas, or nodes, that cannot be trivially obtained by looking at their connectivity alone [3,4]. Notably, the use of controllability metrics derived from Gramian-based energy minimization would enable to identify the best nodal drivers to physically control brain networks [5–7].

Despite initial enthusiasm due to the elegant mathematical formulation and to the promise to address long-standing questions in systems neuroscience [8–10], network controllability suffers from important numerical pitfalls that limit its usability [11–13]. Identifying the driver nodes and the control signals required to effectively guide the system toward desired states is hampered by the curse of dimensionality. This occurs when the number of driver nodes is significantly smaller than the overall size of the network, resulting in outcomes that are challenging to interpret and potentially unreliable [14]. Targeting specific parts of the system is perhaps the most intuitive way to lower the imbalance between the number of drivers and the size of the network to control [15,16]. In addition, using stepwise algorithms to calculate the node control centrality can provide some benefit on the overall accuracy [17]. Yet, those approaches represent *ad-hoc* solutions and fail to tackle the issue in a more fundamentally grounded manner.

By controlling aggregated components of the original network state, output controllability offers a more coherent approach to reduce the problem of dimensionality [18]. For example, controlling the averaged state of the nodes within groups, or modules, has been demonstrated to be an effective strategy to improve accuracy [19]. However, average-state controllability is an over-approximation of the real dynamics as nodes in the same module might have very different functional states. Because network dynamics are constrained by the underlying structure [20–22], we hypothesized that exploiting the actual connectivity would give more representative aggregation schemes and lead to better accuracy.

To this end, we introduced a framework that leverages spectral graph theory to embed the original network state, or signal, into a few representative components that inform how it is represented at different topologically spatial scales [23]. We formally introduced our computational approach for linear-time invariant systems and evaluated its performance in contrast to standard controllability approaches by performing extensive simulations on synthetic networks. By projecting the Gramian matrix into a low-dimensional space we eventually devised parsimonious control centrality metrics to accurately quantify the network driver-target relationships.

We experimentally validated our low-dimensional controllability framework on N = 6134 human connectomes derived from diffusion weighting imaging data to identify the most influential areas of the human connectome, provide a map of the causal interactions between functional brain systems, and characterize the structural hemispheric lateralization.

## Results

We considered unweighted and undirected networks whose dynamics are governed by the following linear time-invariant equation


$$\begin{array}{c} \;\dot{x}\left(t\right)=Ax\left(t\right)+Bu\left(t\right)\\ y\left(t\right)=Cx\left(t\right) \end{array}$$ (1)

Where $x\in R^{n}$ contains the states of the *n* nodes, $A\in R^{n\times n}$ is a stabilized version of the network adjacency matrix *G* and *B* is an arbitrary *n × n*_*d*_ binary matrix directing the external input $u\in R^{n_{d}\;}$ into *n*_*d*_ *≤ n* distinct selected driver nodes. Here, $y\in R^{r}$ with *r < n* represents the low-dimensional output state obtained by projecting the entire state vector *x* into fewer components via the so-called output matrix $C\in R^{r\times n}\;$[24,25].

**Fig 1:**
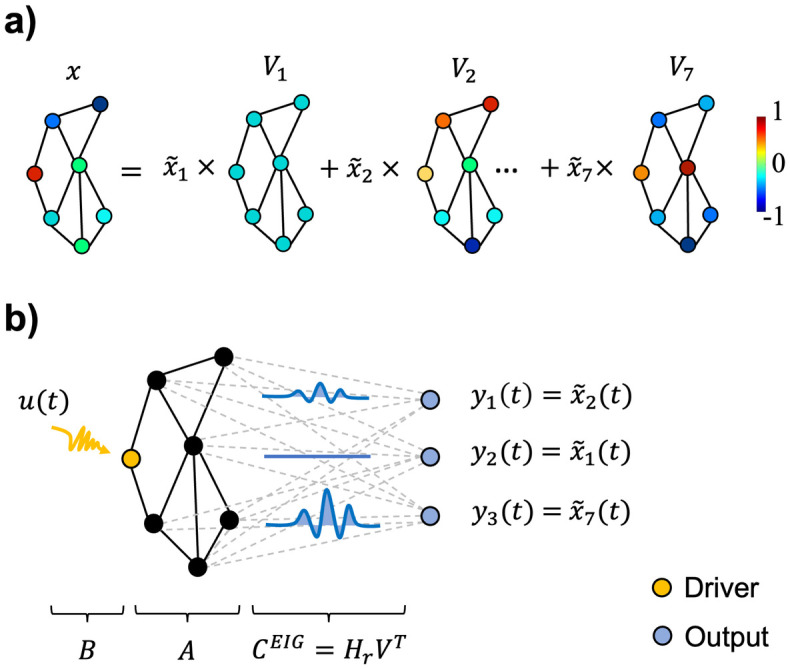
Principles of low-dimensional network controllability. a) Toy network with *n* = 7 nodes. Its state *x* = [*x*_1_,*x*_2_,…,*x*_*7*_] can be seen as a signal over a graph. By exploiting the Laplacian eigenvectors *V* = [*V*_1_,*V*_2_,…,*V*_*7*_] of the network, the signal *x* can be embedded in a spectral space via the graph Fourier transform (*GFT*) $\tilde{x\;}=V^{T}x$. The resulting eigenstate $\tilde{x\;}$ measures how the signal is spatially distributed across different topological scales, from coarser (${\tilde{x\;}}_{1},\;{\tilde{x\;}}_{2},\ldots$) to finer-grained ($\ldots ,{\tilde{x\;}}_{6,\;}{\tilde{x\;}}_{7}$) ones. We can also define the inverse operation *iGFT $x=V\tilde{x\;}$* that is illustrated in this panel. b) Low-dimensional controllability in terms of linear-time invariant (LTI) network output control. Instead of focusing on the network state, the goal is to determine the input signal *u*(t) that steers a low-dimensional output given by the subset of the eigenstate $y\left(t\right)=H_{r}\tilde{x}\left(t\right){=C}^{EIG}x(t)$, where the output matrix *C*^*EIG*^ *= H*_*r*_*V*^*T*^ is obtained by selecting and reordering a number *r* < *n* of spectral components via a filtering matrix *H*_*r*_. In this example, the network has *n* = 7 nodes and the first *r* = 3 spectral components are selected and reordered arbitrarily. Here, the filtering matrix *H*_*r*_ is a 3×7 matrix whose elements are {*h*_1,2_} = {*h*_2,1_} = {*h*_3,7_} = 1 and zero elsewhere.

In general, different configurations for the output matrix can be chosen to aggregate the original network state into fewer components. Instead of selecting *C* in an arbitrary or predetermined fashion, we derived it directly from the network structure by considering the Laplacian linear operator *L = D−A*, where *D* is a diagonal matrix containing the node degree sequence [23]. By construction, *L* can be decomposed into several eigenvectors *V* = [*V*_1_,*V*_2_,…,*V*_*n*_] or eigenmaps, associated with incrementing eigenvalues *λ*_*i*_ that inform on the network structure load from coarser to finer-grained topological scales. Eigenmaps serve as a basis to derive a spectral representation of the network state $\tilde{x}=V^{T}x$, here referred to as *eigenstate* (Fig 1a). By selecting a smaller number of eigenmaps, we aimed to control the low-dimensional eigenstate


$$y\left(t\right)=H_{r}\tilde{x}\left(t\right)=C^{EIG}x\left(t\right)$$ (2)

where the output matrix *C*^*EIG*^ *= H*_*r*_*V*^*T*^ is obtained by selecting and reordering a number *r < n* of eigenmaps via an arbitrary filtering matrix *H*_*r*_ (Fig 1b).

### Reducing the number of eigenmaps improves control precision

**Fig 2:**
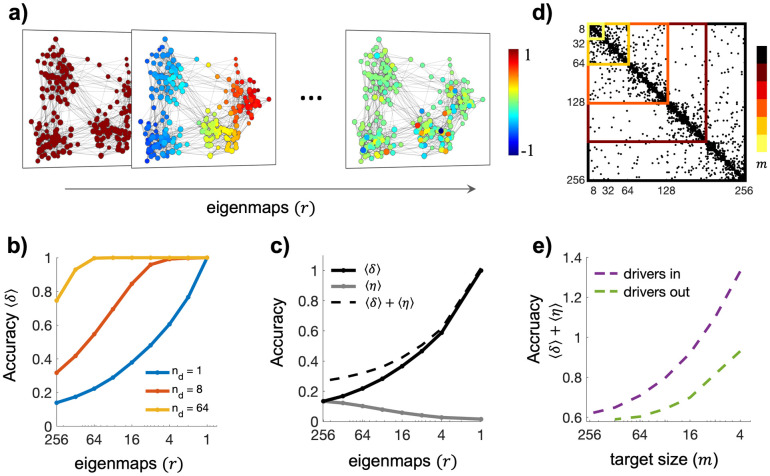
Fundamental advantage of low-dimensional controllability. a) Laplacian eigenvectors (or eigenmaps) for one realization of the hierarchical modular small-world network (HSWM). Colors identify the different spatial contributions of the nodes at increasingly finer topological scales. b) Control accuracy in terms of precision (*δ*) as a function of the number of eigenmaps. Different lines correspond to different number of drivers. Candidate drivers are progressively added according to their betweenness centrality values in a descending order. Results correspond to the mean values obtained from 100 simulated networks. c) Average node precision $\left\langle \delta \right\rangle =\frac{1}{n}\sum_{i=1}^{n}\delta_{i}$ (black) and representativeness$\;\left\langle \eta \right\rangle =\frac{1}{n}\sum_{i=1}^{n}\eta_{i}$ (grey) for single-driver control as a function of the number of eigenmaps. Dashed curves correspond to the total accuracy⟨*δ*⟩+⟨*η*⟩. Results correspond to the mean values obtained from 100 simulated networks. d) Schematic representation of a hierarchical modular small-world network (HSWM) whose target set is progressively expanded by including an increasing number of nodes *m*. The inclusion criterion starts with the nodes in one module and then continues by considering the nodes in the subsequent modules until the covering of the entire network. e) Average node total control accuracy ⟨*δ*⟩+⟨*η*⟩ for single-drivers as a function of the target size *m*. Magenta/green curves correspond to results obtained by averaging the total accuracy for the drivers inside/outside the target. Results correspond to the mean values obtained from 100 simulated networks.

We considered hierarchical modular small-world (HMSW) networks whose Laplacian eigenmaps informed on the network partition across multiple scales [26] (Fig 2a, Materials and Methods). Without loss of generality [27], we set the initial network state in the origin $x_{0}=\vec{0}\in R^{n}$ and the final state as a random departure from it $x_{f}\sim N\left(\mu_{f}=1,\sigma_{f}=10\right)\in R^{n}$, which corresponded to steering the related eigenstate from $y_{0}=\vec{0}$ to *y*_*f*_ = *C*^*EIG*^*x*_*f*_. Candidate drivers were chosen by ranking the nodes according to their betweenness centrality so as to ensure homogeneous distances from the rest of the network [28]. We then computed the associated input signals *u*(*t*) by minimizing the output cost function


$$J_{\rho }(u,t_{f})=\;∥y_{f}-y\left(t_{f}\right){∥}^{2}+\rho \int_{0}^{t_{f}}∥u\left(\tau \right){∥}^{2}d\tau$$ (3)

where *ρ* is a regularization parameter balancing the accuracy of the solution with respect to the signal energy [29]. Note, that this formulation imposes soft constraints on the output states by minimizing the distance with the desired configuration only at the final time *t*_*f*_ and gives more flexibility compared to hard-constrained versions which minimize the distance along the entire time horizon [30] (S1 Text). By injecting the optimal inputs back into the model dynamics, we considered the cosine similarity to measure the control accuracy in terms of *precision $\;\delta =\frac{y\left(t_{f}\right)\;∙\;y_{f}}{∥y\left(t_{f}\right)∥∥y_{f}∥}$*. To ensure sufficiently accurate solutions we selected the following parameter values, = 10^−4^, *t*_*f*_ *=* 1 (S1a Fig) and time resolution *dτ* = 0.01 (S2 Fig). To evaluate the impact of the low-dimensional control on the original network state, we also measured a secondary metric, namely the *representativeness* $\eta =\frac{x(t_{f})\;∙\;x_{f}}{∥x(t_{f})∥∥x_{f}∥}$.

Results showed that reducing the number of eigenmaps *r* significantly increases the control precision regardless of the number of drivers (one-way ANOVAs *F >* 1048.22, *p<*10^−32^, Fig 2b). The most striking benefit occurs for single-driver control, which is very common in real-world scenarios. As a side-effect the representativeness decreases with *r*. Evaluating optimal tradeoffs such as the total accuracy *δ + η* can therefore inform on how to choose the number of eigenmaps (Fig 2c). We next focused on target network control, which aims at controlling a subnetwork *S* with *m ≤ n* nodes (Fig 2d). To this end, we computed the Laplacian considering the internal connectivity of S (Materials and Methods). As in full network control, we calculated the related low-dimensional eigenstates and measured the total accuracy as a function of the target size. Results showed an overall benefit in controlling smaller parts of the network, especially when considering internal drivers and using a relatively low number of eigenmaps (Fig 2e).

A key parameter affecting the performance of the low-dimensional approach is the heterogeneity of the network nodes’ states. In a separate analysis, we showed that the higher the standard deviation of the final state *x*_*f*_ the lower the control precision and representativeness. This effect can only be compensated in terms of precision by further reducing the number of eigenmaps (S1b Fig). All these findings were obtained by selectively including eigenmaps from the most to the least informative in terms of *y*_*i*_ magnitude. Changing the inclusion criterion, such as ranking them according to the *λ*_*i*_ values, led to similar tendencies in terms of precision but lower representativeness when the final state becomes highly dispersed, i.e. *σ*_*f*_*>*1 (S1c Fig). In addition, the trends stayed relatively similar when considering networks with different topologies, i.e., random and scale-free (S1d Fig) or when the nodes’ functional states were assigned according to processes that are not Gaussian, such as transitioning from an initial uniform distribution to a final modular organization, the latter typically observed in several brain systems [31,32] (S3 Fig). Finally, although larger network sizes reduce control precision and increased input energy, these effects can be mitigated by selecting fewer eigenmaps (S4 Fig).

**Fig 3:**
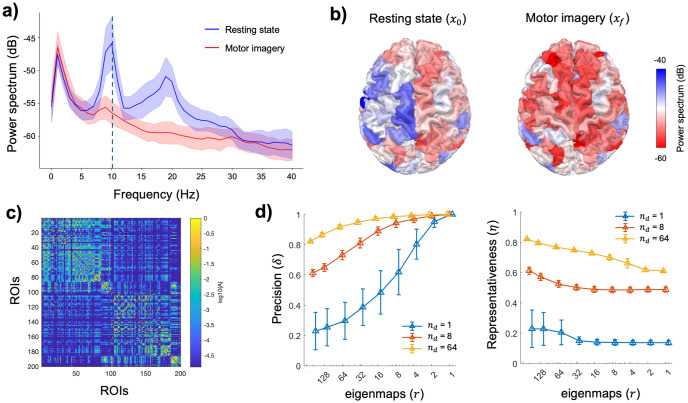
Application to actual brain network functional states. a) Power spectrum for the left somatomotor area contralateral to the imagined movement. Blue = motor imagery, red = resting state. Solid lines = average across blocks, shaded bands = standard deviation. The vertical dashed line spots out the values at 10 Hz extracted for all the regions of interest (ROIs=nodes) and used for the controllability analysis. b) Spatial distribution of the trial-averaged power spectrum at 10 Hz for the initial resting state and the final motor imagery state. The brain, viewed from above, frontal lobe upside, is obtained from the real MRI of the subject. Power spectrum density (PSD) values are here reported in decibel and show the typical motor-related power decrease occurring in the left somatomotor regions contralateral to the right-hand movement. For the controllability analysis, PSD values have been normalized so that *μ*_0_ = 0, *σ*_0_ = 9.27 for the resting state and *μ*_*f*_ = −5.33, *σ*_*f*_ *=* 4.59 for the motor imagery state. c) Connectivity matrix of the structural connectome obtained from the DTI data of the subject. The top-left block corresponds to the left hemisphere, while the bottom-right block corresponds to the right hemisphere. The links weights measure the number of axonal fascicles between ROIs and are reported in logarithmic scale for the sake of readability. The driver node, selected according to the highest between centrality, is the somatomotor area in the right hemisphere. d) Block-averaged control precision and representativeness for the real brain network data as function of the number of eigenmaps. The target is the entire network, i.e. *m* = *n* = 200. Input control signals are obtained solving Eq 3 using the parameters *t*_*f*_ = 1, *dτ =* 0.01, and *ρ =* 0.0043, 0.0121, 0.0234 respectively for one, eight and 64 drivers. Vertical bars denote standard deviations.

To gain a deeper understanding of the interpretation and effects on practical applications, we eventually examined functional network states derived from actual experimental neuroimaging data. Specifically, we considered source-reconstructed EEG activity recorded in a stroke patient involved in a typical brain-computer interface task as well as the structural connectome derived from DTI data (Fig 3a–3c, Materials and Methods). In the framework of our study, our goal was to assess the difficulty of steering the brain from a resting state to a motor imagery state involving the affected hand. Results showed that globally the control accuracy follows the same behavior already observed in Fig 2. Using a reduced number of eigenmaps allowed to retrieve a high precision, especially in the extreme condition where there is only one driver, and the representativeness is low (Fig 3d). Notably, by using the typical interareal axonal conduction delay [33], we could provide an approximate estimate of the physical units for the time horizon needed to steer the brain by means of an external control input, i.e. 0.1<*t*_*f*_<0.3 seconds (S5 Fig).

### Low-dimensional controllability of brain systems

To quantify the ability of individual brain regions to influence the activity of other areas, we introduced a control metric based on a low-dimensional projection of the Gramian matrix [5]


$$W=\int_{0}^{\infty }e^{A\tau }BB^{T}e^{A^{T}\tau }\;d\tau$$ (4)

where *B* selects the i^th^ candidate driver. Like Eq 2, we operated such projection via the *C*^*EIG*^ matrix, i.e., $W^{EIG}=C^{EIG}W{C^{EIG}}^{T}$. Its smallest eigenvalue $\lambda_{min}^{EIG}$ gave a low-dimensional version of the worst-case control centrality *λ*_*min*_, measuring the amount of energy needed for node *i* to steer the network into the most difficult-to-reach state [34].

**Fig 4:**
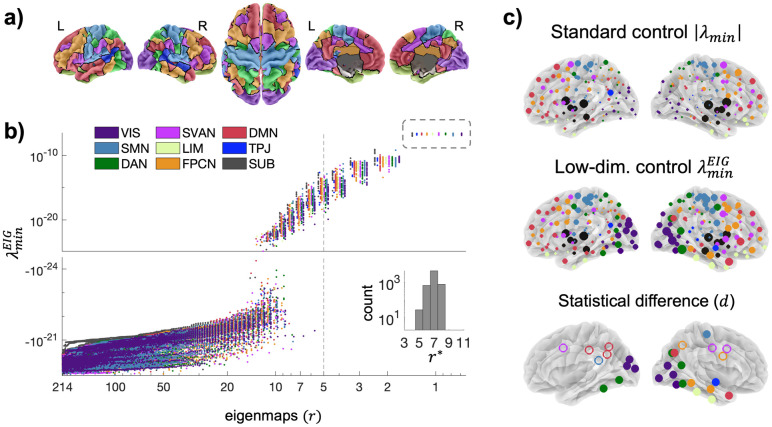
Single-driver controllability of brain networks. a) The Yeo2011 brain atlas parcellation. Each of the 214 regions of interests (ROIs) are organized in 9 functional systems: the visual network VIS, the somatomotor network SMN, the dorsal attention network DAN, the saliency and ventral attention network SVAN, the limbic network LIM, the frontoparietal control network FPCN, the default mode network DMN, the temporoparietal junction TPJ, and the subcortical network SUB. b) Low-dimensional worst-case control centrality $\lambda_{min}^{EIG}$ values as a function of the number of eigenmaps *r*. Each point corresponds to a different node (ROI) controlling the entire brain. Colors code for different systems. Values are shown for a representative subject. By decreasing *r* all $\lambda_{min}^{EIG}$ values become positive and numerically reliable after a critical threshold r*. The inset illustrates the distribution of r* from all subjects (N = 6134). Note that the standard metric *λ*_*min*_ (*r* = *n* = 214) gives the lowest negative values making it difficult to interpret. c) Group-averaged spatial distribution of standard (|*λ*_*min*_|) and low-dimensional ($\lambda_{min}^{EIG}$) control centrality. Low-dimensional control centrality exhibits a significant reorganization compared to standard control centrality. The third row shows the ROIs that significantly gain (filled circles) or lose (empty circles) importance as compared to standard control (Sign test *p*≪ 10^*−*6^, Cohen’ |*d*|>0.5, S2 File).

We considered structurally weighted brain networks obtained from healthy human subjects in the UK-biobank cohort (www.ukbiobank.ac.uk). Brain networks consisted of *n* = 214 nodes including cortical and subcortical regions of the Schaefer atlas [35] (S1 File). They were grouped into nine differently specialized functional systems [36]: the visual (VIS), the somatomotor (SMN), the dorsal attention network (DAN), the saliency and ventral attention network (SVAN), the limbic (LIM), the frontoparietal control network (FPCN), the default mode network (DMN), the temporoparietal junction (TPJ), and the subcortical network (SUB) (Fig 4a, Materials and Methods). Our first main result showed that reducing the number of eigenmaps allows to retrieve reliable controllability metrics (i.e., $\lambda_{min}^{EIG}>0)$, whereas this failed when using standard *λ*_*min*_. Achieving control over low-dimensional projections of the brain network became feasible for every node when r*≤ 5. Such a transition threshold is in line with previous evidence [14], and we used it in all our subsequent analyses (Fig 4b). Compared to standard worst-case controllability some regions exhibited similar relative spatial contributions (e.g. SUB), while others faded out or gained importance notably in the visual-temporal part of the right hemisphere (e.g. VIS, DAN) (Fig 4c).

**Fig 5:**
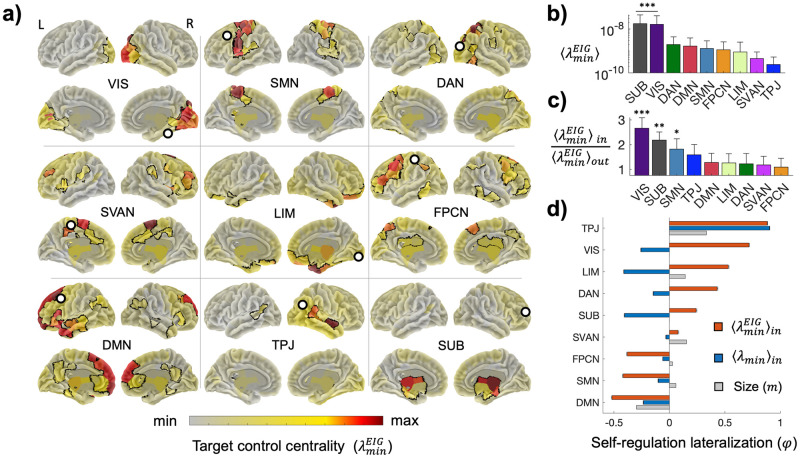
Target controllability of brain systems. a) Spatial distribution of group-averaged control centrality $\lambda_{min}^{EIG}$ when targeting each separate brain system. Target systems are contoured by black curves. Best drivers tend to fall within each target as indicated by the colorbar. White circles identify the best drivers outside the target (Tab 1). For each system results are illustrated for left (L) and right (R) hemisphere in both ventral (up) and dorsal (bottom) views. b) System controllability $\langle \lambda_{min}^{EIG}\rangle$ as the mean control centrality of all the nodes targeting a specific system. Bars indicate group-averaged values and error bars standard deviations. Asterisks denote the systems whose controllability is significantly higher according to a post-hoc ANOVA analysis (*p* ≪ 10^−6^). The more the asterisks, the stronger the difference. Tukey-HSD Post-hoc ***p ≪ 10^−6^, **p < 0.001,*p < 0.05 (S2 File). c) Ratio between self-regulation ${\langle \lambda_{min}^{EIG}\rangle }_{in}$ and external regulation ${\langle \lambda_{min}^{EIG}\rangle }_{out}$ measured respectively by the mean control centrality of the nodes inside and outside a specific targeted system. Bars indicate group-averaged values and error bars standard deviations. Values have been log-transformed for the sake of readability. Asterisks denote the systems whose controllability ratio is significantly higher according to a post-hoc ANOVA analysis (*p* ≪ 10^−6^). The more the asterisks, the stronger the difference. Tukey-HSD Post-hoc ***p ≪ 10^−6^, **p < 0.001,* *p <* 0.05 (S2 File). d) System lateralization in termsof self-regulation from the right (R) and left (L) hemisphere $\varphi =\frac{\zeta^{R}-\zeta^{L}}{\zeta^{R}+\zeta^{L}}$. Red colors correspond to low-dimensional controllability $\zeta ={\langle \lambda_{min}^{EIG}\rangle }_{in}$.Blue colors correspond to standard controllability $=\;{\langle \lambda_{min}\rangle }_{in}$. Grey colors show the lateralization in terms of number of nodes of the systems in each hemisphere. Bars indicate group-averaged values and errorbars standard error means. Lateralization of low-dim. self-regulation significantly depends on the brain system (ANOVA, *p* ≪ 10^−6^).

Using the target controllability approach described in the previous section, we next considered the Laplacian of the subnetworks corresponding to the brain systems and evaluated their controllability (S6 Fig). Results pooled from all subjects showed that systems could be controlled from both internal and external nodes enabling to derive optimal driver-target configurations (Fig 5a and Table 1). More in general, we reported a system controllability hierarchy with subcortical and visual systems among the easiest to control (Fig 5b). Internal drivers exhibited higher control centrality compared to external ones, indicating a preferential system self-regulation with respect to external regulation (Materials and Methods). That was particularly evident for SUB, but also for the primary visual and somatomotor system, the latter to a lesser extent (Fig 5c). This result was not necessarily due to the spatial proximity between the driver and the target, but rather to the topological distance in terms of shortest paths (S7 fig).

**Table 1:** Driver-target best configurations.

Target system	Inside driver	Outside driver
VIS	VisPeri_ExStrSup_3-R	LimbicA_TempPole_4-R
SMN	SomMotB_Cent_1-L	ContB_PFCl_1-L
DAN	DorsAttnA_SPL_4-R	VisCent_ExStr_5-R
SVAN	SalVentAttnA_FrMed_2-R	SomMotA_7-L
LIM	LimbicA_TempPole_1-R	VisCent_Striate_1-R
FPCN	ContA_IPS_1-L	SomMotA_7-L
DMN	DefaultA_PFCd_1-L	ContB_PFCl_1-L
TPJ	TempPar_1-R	DefaultC_IPL_1-R
SUB	Sub_thalamusproper-R	DefaultA_PFCm_3-R

Drivers are selected according to their maximum group-averaged low-dimensional control centrality $\lambda_{min}^{EIG}$.

We then investigated the systems lateralization in terms of self-regulation to accumulate in one or the other hemisphere. For each targeted system, we computed a lateralization index$\varphi =\frac{\zeta^{R}-\zeta^{L}}{\zeta^{R}+\zeta^{L}}$, where $\zeta ={\langle \lambda_{min}^{EIG}\rangle }_{in}$ for the nodes in the right (R) or left (L) hemisphere. Resultsshowed significant system-dependent lateralization, which was not merely due to possible differences between hemispheres in terms of system size (Fig 5d). Such lateralization was perfectly in line with the known functional experimental evidence, such as the right-dominated limbic and dorsal-attention network and could not be retrieved when using the standard worst-case control metric *λ*_*min*_.

### Mapping inter-system controllability in the brain

**Fig 6:**
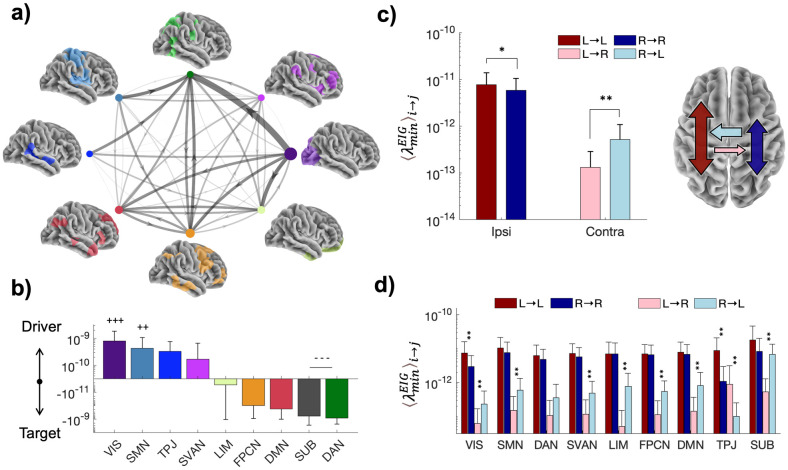
Control relationships between brain systems. a) The group-averaged controllability meta-graph. Nodes correspond to different brain systems. Directed weighted links illustrate the group-averaged geometric mean of the control centrality for system *i* when targeting system *j ${\langle \lambda_{min}^{EIG}\rangle }_{i\to j}$*. The darker and thicker the link, the stronger the influence of system *i* on *j*. Self-loops and the SUB network are not represented as their control centrality is several orders of magnitudes higher. b) System control unbalance as the difference between the sum of outgoing and incoming weighted links from the individual meta-graphs. Positive values = tendency to act as driver. Negative value = tendency to act as target. Bars indicate group-averaged values and errorbars standard deviations. +/- denote the systems whose controllability is significantly higher/lower according to a post-hoc ANOVA analysis (*p* ≪ 10^−6^). The more the symbols, the stronger the differences. Tukey-HSD Post-hoc ***p ≪ 10^−6^, **p < 0.001 (S2 File). c) Hemispheric preference in terms of ipsilateral and contralateral control capacity. Dark colors denote *ipsilateral* control as the mean of the nodes in hemisphere *i* targeting the same hemisphere ${\langle \lambda_{min}^{EIG}\rangle }_{i\to i}$. Light colors indicate *contralateral* control as the mean of the nodes in hemisphere *i* targeting the other hemisphere ${\langle \lambda_{min}^{EIG}\rangle }_{i\to j}$. Bars indicate group-averaged values and errorbars standard deviations. L = left hemisphere, R = right hemisphere. Asterisks indicate Cohen’s *d* values measuring effect sizes. Sign test **p ≪ 10^−6^,Cohen’ |*d*|>0.5, *p ≪ 10^−6^, Cohen’ |*d*|>0.2, S2 File). d) Hemispheric preference of single systems in terms of their ability to control the entire ipsilateral and contralateral hemisphere. Same graphical conventions as before.

To better understand how different systems were influencing each other we next studied their reciprocal role as drivers and targets. For each pair of systems *i* and *j* we established a directed weighted link by calculating the mean control centrality of the nodes in *i* when targeting *j*, i.e., *${\langle \lambda_{min}^{EIG}\rangle }_{i\to j}$*. By inspecting the resulting meta-graph, we found a heterogeneous strength and distribution of causal influences that enabled to identify the preferential targets and drivers for each system (Fig 6a). Notably, primary systems exerted a strong controlling influence over the dorsal-attentional network (VIS→DAN, SMN→DAN), while more balanced interactions were found between associative systems (e.g., FPCN↔DMN, DMN↔LIM).

To quantify the control unbalance, we computed for each system the difference between the outgoing and incoming sum of the weighted links of the meta-graph. Results confirmed that both VIS and SMN exhibit a significantly higher tendency to control as compared to SUB and DAN which instead appeared much easier to be controlled (Fig 6b). A more balanced profile was instead observed for the other systems, such as the limbic system. Similar results were also obtained when using a finer-grained version of the Yeo2011 parcellation (S8 Fig). We finally investigated hemispheric dominance in terms of control capacity. To do so, we computed the $\lambda_{min}^{EIG}$ value of each node when targeting the two hemispheres, separately. We defined *i)* the *ipsilateral* control as the mean ${\langle \lambda_{min}^{EIG}\rangle }_{i\to i}$ of the nodes in one hemisphere targeting the same hemisphere, and *ii)* the *contralateral* control as the mean ${\langle \lambda_{min}^{EIG}\rangle }_{i\to j}$ of the same nodes targeting the other hemisphere.

Taken separately, each hemisphere exhibited a stronger capacity to control itself in contrast to its ability to influence the other. However, non-trivial results emerged when comparing the two hemispheres. While the ipsilateral control of the left hemisphere (L➔L) was significantly higher than the right hemisphere (R➔R), the contralateral control of the right hemisphere (R➔L) was significantly higher than the left hemisphere (L➔R) (Fig 6c). This global behavior could be explained by the same tendency across all the local brain systems, but the temporoparietal junction whose contralateral control exhibited an opposite trend (Fig 6d). Altogether, these findings indicate that the left hemisphere exhibits better self-regulation, while the right hemisphere has a stronger ability to influence its counterpart.

## Discussion

### Spectral graph theory and network controllability

Spectral graph theory (SGT) is a powerful and versatile framework for understanding and analyzing complex interconnected systems. SGT is particularly concerned with the eigenvalues and eigenvectors of the Laplacian matrix associated with a graph and informs on the global and finer-grained connectivity structures in the network [37,38]. Laplacian eigenvectors, also known as eigenmaps, find various applications in network science, such as data clustering and dimensionality reduction via spectral embedding [39]. From a broader graph signal processing angle, the Laplacian eigenmaps technique informs on dynamical processes like synchronization or diffusion and is a suitable framework to analyze how external perturbations propagate in a network [21]. More recently, SGT has been developed to identify connectivity gradients in the brain [40], disentangle brain structure and function [41], improve machine learning for systems neuroscience [42], and decompose brain dynamics into graph Fourier modes [43,44].

Despite its potential to reduce the complexity of signals on graphs, SGT has been poorly explored in the context of network controllability. Network controllability provides a mathematically grounded framework to identify drivers, influence network dynamics and understand causal relationships, but it still faces critical challenges that hamper its usability in many practical situations [5,45]. This is mainly due to the presence of ill-posed conditions that make the problem numerically unstable with important consequences on the solutions, such as an overestimation of the drivers or completely unreliable input-controlling signals [15]. Hence, the development of methods to reduce the problem complexity is paramount to enhance the usability and interpretation of network controllability in real-world applications. Targeting specific subnetworks [15–17] or reducing the network to fewer averaged nodes [19, 46] have shown substantial benefits, yet they represent approximations that oversimplify the original network structure.

SGT offers a more flexible solution to reduce problem complexity enabling to write the network states as a linear combination of its actual connectivity structures (the eigenmaps) across different topological scales. By establishing a link between SGT and output controllability, we showed that controlling the most representative spectral components from the graph Fourier transform, rather than considering the entire original network state, leads to significant improvements even in the presence of unbalanced ndm≪1 and noisy configurations. The price to pay for such a control precision consists of an almost equivalent loss in the representativeness of the solution in terms of capacity to reproduce the original network state. Optimal trade-offs can be further investigated in future research to better capture the interplay between network topology, the number of output components, and the smoothness of the state solution.

### Theoretical and effective controllability of brain networks

Network controllability can provide insights into how different regions of the brain interact to perform various cognitive and motor functions by passing electrical activity through white matter connections [2,47]. Its theoretical framework can be used to experimentally test basic questions in network neuroscience and inform noninvasive brain stimulation or neurofeedback approaches to enhance cognitive functions, such as memory and learning [48]. Furthermore, investigating the controllability of brain networks can shed light on the causal mechanisms underlying neurological and psychiatric disorders [49] as well as help identify potential entry points for therapeutic interventions, such as brain stimulation techniques for conditions like epilepsy, Parkinson’s disease, stroke, and chronic pain [50].

Despite its potential, brain network controllability faces a few methodological challenges that limit its usability. This is mainly due to the unreliability of control centrality metrics, such as the worst-case control *λ*_*min*_, which are mathematically correct but have little translational value when the number of drivers is much lower than the nodes to control. The main consequence is that is not always possible to infer the control power of single brain areas because it cannot be assessed numerically and remains difficult to interpre [12–14]. Here, we showed that low-dimensional controllability allows overcoming this limitation and reveals previously unappreciated brain drivers in the visual and dorsal attention network that could not be identified by merely looking at their connectivity strength (Figs 4 and S9). SUB is the most controllable system possibly due to its rich-club organization integrating information from remote parts of the brain [51], and together with VIS and SMN it exhibits a high tendency to self-regulate as compared to other associative systems [52].

The analysis of the self-regulation allowed to clarify functional [53] and anatomical [54] hemispheric asymmetries such as the right preference for TPJ and SVAN -partially overlapping with the ventral attention network- as well as for LIM, in line with the control of emotional processing [55]. In addition, the found left-hemisphere advantage of SMN and DMN aligned with the suggested preferential role in action selection [56] and endogenously generated cognition [57]. More importantly, we provided new insights for those systems whose asymmetry is more controversial such as VIS [58] and DAN [59,60]. From a control-theoretic perspective, these systems exhibit a strong tendency to self-regulate via the right hemisphere and provide an anatomical basis for the common clinical observation that spatial neglect is more frequent, persistent, and severe after damage to the right hemisphere as compared to lesions to the left homotopic areas [61].

### Causal relationships between brain systems

The presence of several systems, often called “networks”, coexisting in the brain is an emerging feature of its intrinsic functioning and crucial to understanding human behavior in both healthy and pathological conditions [53]. By leveraging connectomics to trace functional connectivity, several large-scale brain systems have been identified that are important for decision making (FPCN), emotion, motivation and memory (LIM), attention for externally-directed tasks (DAN), introspective thoughts (DMN), visual and somatosensory processing (VIS, SMN), and internal/external thinking (SVAN) [62]. Notably, these systems exhibit a hierarchy to their operation, but they do not work in isolation. Instead, they are known to integrate and synchronize to carry out complex functions [36,63].

At a larger scale, the brain hemispheres can also be considered as anatomically separated systems that functionally interact to accomplish complex tasks [64–66]. Cerebral specialization is not only important to underpin the neural basis of language, attention, and motor control, but it is also critical to assess and treat related deficits due to mental or neurological disorders [64,67]. Yet, determining how large-scale systems simultaneously and causally influence each other is still poorly understood. Here, we showed that drivers in the primary systems (VIS, SMN) are particularly apt to influence distributed activity in associative and attentional systems (e.g., DAN, SVAN), while drivers in the prefrontal cortex exhibited facilitated control of SUB, SMN, and DMN (Figs 5 and 6). This result was further confirmed by the meta-wiring diagram showing how brain systems simultaneously interact from a control theoretic perspective.

The role of primary systems as controlling drivers and that of associative systems as controlled targets, matched previous results obtained with network communication models of brain cognition [68] and aligned with theories postulating that brain topography is organized along spatial and functional gradients [69,70]. These gradients span from peripheral regions that handle perception and action to core areas dealing with more abstract cognitive functions. Our findings revealed that core attentional networks are strongly controlled by primary peripheral systems, which are instead less prone to be endogenously influenced due to their relatively higher dependency from external events and stimuli. In terms of hemispheric asymmetries, our results extend recent evidence using fMRI functional connectivity suggesting a preference for the left hemisphere to interact with itself and for the right hemisphere to integrate information both locally and from the other hemisphere [71]. Our findings offer an anatomical basis for this fundamental result by unveiling unbalanced directed influences within and between hemispheres.

### Perspectives and limitations

By leveraging principles from spectral graph theory, the introduced low-dimensional controllability framework offers a reliable resource to interrogate, influence and ultimately understand the behavior of complex biological systems.

In terms of concrete applications, brain stimulation represents a promising one. Brain stimulation uses electrical or magnetic impulses to influence neural activity, often for neurological or psychiatric treatment. In open-loop settings, the stimulation of the hotspot is fixed while in closed-loop ones it adapts to brain activity in real time, offering more personalized and responsive treatment [72–74]. In both situations, it is crucial to minimize the stimulation energy to ensure the safety and comfort of the subject and to avoid side effects like fatigue or habituation. To this end, the results on control centrality can guide the selection of the optimal driver nodes (i.e. the hotspots) to ensure efficient outcomes with minimal energy expenditure (Fig 5). In addition, by looking at how brain systems influence each other from a control perspective, one can predict collateral effects of focal stimulations and refine neurostimulation strategies (Fig 6).

Finally, it is important to remember that the presented approach assumes the linearity of the system and the absence of stochasticity. While linear models don’t capture the complexity of most neural processes, they can locally approximate the related non-linear dynamics and provide an easier interpretation of the results [47, 75], particularly at macroscales [76]. By assuming that the signal propagation properties are deterministically modeled, one indeed ignores the role of noise on neural signals on both small and large timescales [77,78]. However, it is common practice to first focus on key model features that are independent of noise [79], notably in the context of network controllability [80]. For more in-depth discussion and details about the topic, we refer to the above-mentioned articles and reviews.

## Materials & methods

### Ethics statement

The patient, whose processed data have been used in Fig 3, gave written informed consent for participation in the study, which was approved by ethical committee of Pitié-Salpêtrière Hospital (CPP 2019.01.05 bis _18.12.05.60556).

### Laplacian of stabilized networks

The graph Laplacian *L = D−A* is a symmetric matrix that can be rewritten via singular value decomposition as *L* = *V*^*T*^ Λ*V*, where Λ contains its increasing eigenvalues (*λ*_1_≤*λ*_2_…≤*λ*_*n*_) and *V =* [*V*_1,_*V*_2_,…,*V*_*n*_] are the associated eigenvectors [23]. By construction, eigenvectors are orthonormal (i.e., *V*^*T*^ *V = I*) and constitute a projection basis to obtain the spectral representation of any signal *x* on a graph via the so-called Graph Fourier Transform (GFT) [23], i.e., *${\tilde{x}=V}^{T}x$*. Here, networks correspond to graphs whose adjacency matrices *G* code for the presence/weight of links between the nodes. To ensure the stability of the system and the existence of the Gramian we next added negative self-loops to the network via a linear transformation *A* = *G-cI*, with *c* = *λ*_*max*_(*G*)+*ϵ*, where *ϵ =* 2 × 10^−16^ to ensure that the spectrum of A is strictly negative [24,30]. It can be easily demonstrated that such transformation does not alter the network Laplacian which is a linear operator (S1 Text).

### Target network control

Let *S* = [*s*_1_,s_2_,…,*s*_*m*_] be a subset of *m < n* target nodes indexed by *s*_*i*_∈ [1,*n*] and *G*(*S*,*S*) the corresponding adjacency matrix. We formulated the related target control problem by considering the Laplacian of the subnetwork *L*_*S*_ = *D*_*S*_−*G*(*S*,*S*), where *D*_*S*_ is a diagonal matrix that contains the degree sequence of the target nodes excluding the links to the rest of the network. Note that a precise relation holds with the submatrix of the original Laplacian *L*(*S*,*S*) = *L*_*S*_*+Z*_*S*_, where *Z*_*S*_ is a diagonal matrix containing the sum of the links connecting the target nodes to the rest of the network [81]. Hence, *L*_*S*_ exactly matches the actual spatial topological scales of the subnetwork and it is not biased by external influences. By considering the state of the subnetwork *x*_*S*_(*t*) and the related eigenmaps *V*_*S*_ we calculated the target *eigenstate* $\tilde{x_{S}}(t)={V_{S}}^{T}x_{S}\left(t\right)$ and derived its low-dimensional output


$$y_{S}^{EIG}\left(t\right)=H_{r}\tilde{x_{S}}\left(t\right)=C_{S}^{EIG}x_{S}\left(t\right)$$ (5)

where the output matrix $C_{S}^{EIG}=H_{r}{V_{S}}^{T}\;$ is obtained by selecting and reordering a number *r* < *m* of spectral components via a filtering matrix *H*_*r*_.

### Hierarchical modular small-world network and controllability metrics

We used the HMSW model [26] to generate synthetic networks with *n* = 256 nodes, 8 = log_2_ (256) hierarchical levels, and an average network density of 0.035. Other parameters were initial cluster size = 2, and connection density fall-off per level = 2.5. The choice of this model and its parameters was particularly relevant for the purposes of this study and has been widely adopted in topological analysis of brain networks [82] which exhibit hierarchical modularity [83].

Starting from the worst-case low-dimensional control centrality $\lambda_{min}^{EIG}$ of each node, we defined aggregated metrics to measure larger topological effects, i.e., i) *system controllability* $\langle \lambda_{min}^{EIG}\rangle$ as the mean of all the nodes targeting a specific subnetwork, ii) *self-regulation* ${\langle \lambda_{min}^{EIG}\rangle }_{in}$ as the mean of all the nodes inside a target subnetwork, and iii) *external regulation ${\langle \lambda_{min}^{EIG}\rangle }_{out}$* as the mean of all the nodes outside a target subnetwork.

### UK-Biobank neuroimaging data and tractography

The UK-biobank dataset is a large-scale cohort containing clinical, genetic, and imaging data [84]. We selected N = 6134 human subjects who had both T1 weighted and diffusion MRI and no known disease history, i.e., international classification of diseases ICD-10 = *‘none’*. Population statistics: 50.73% women, 88.72% right-handed, and age 62.41±7.25 at the time of the MRI scans. Informed consent was obtained from all UK Biobank participants. We rejected those who requested the withdrawal of their data. Procedures are controlled by a dedicated Ethics and Guidance Council (http://www.ukbiobank.ac.uk/ethics), with the Ethics and Governance Framework available at https://www.ukbiobank.ac.uk/media/0xsbmfmw/egf.pdf. IRB approval was also obtained from the North West Multi-centre Research Ethics Committee. This research has been conducted using the UK Biobank Resource under Application Number 53185. Downloaded imaging data were corrected and preprocessed with the UK-biobank pipeline [85].

As for the processing, we computed Tissue response and Fiber Orientation Distribution using multi-tissue and multi-shell algorithms in MRtrix3 [86]. T1 images were aligned to an extracted mean b0 volume via the FLIRT function in FSL [87]. We performed a 5-tissue type segmentation to compute the grey-white matter interface. These were then used in MRtrix3 to compute an anatomically constrained tractography with a cut-off of 0.1 and a density of 1M streamline that was shown to be sufficient for reproducibility [88]. As for the parcellation, we used the Schaefer atlas of 200 cortical regions (i.e., nodes) exhibiting high structural and functional relevance [89]. Its regions were grouped into eight cortical systems according to the Yeo2011 mapping [36]. The cortical atlas was complemented by 14 subcortical regions from the FreeSurfer segmentation [52]. The parcellation was transferred to the subject space using the T1 linear co-registration and the UK-biobank warp field. It was finally dilated and masked to be used in MRtrix3 along the SIFT [90] for the connectome extraction. Fiber assignment was done with a radial search of 3mm and the resulting connectomes were symmetric with a zero diagonal. Link weights correspond to the number of fibers between two nodes.

### Experimental EEG data, source reconstruction, and tractography

Data were collected from a stroke patient suffering from a 1-month subcortical lesion leading to hemiparalysis of the right arm. The electroencephalographic (EEG) experiment was conducted with a 64 EEG-channel system, with active sensors (Easycap, Germany) placed according to the standard 10–10 montage and associated with two BrainAmp amplifiers. EEG signals were referenced to TP10 (closest channel to mastoid), with the ground electrode located in FT9, and impedances were kept lower than 30 kOhms. EEG signals were recorded with a sampling frequency of 500 Hz and a bandwidth of 0.01-250Hz. During the recording session, the patient was comfortably seated in a dimly lit room, with the upper limbs resting on a cushion. The patient was instructed to perform 60 trials of motor imagery and 60 trials of resting states in a randomized fashion. Each trial lasted 4 seconds and the entire session, including the visual stimuli and EEG recording, was handled via the https://openvibe.inria.fr/ software.

An echo-planar imaging at 3T (Siemens) with a standard head coil for signal reception was used for the MRI acquisitions. High-resolution 3-D anatomical MPRAGE images was first acquired. Diffusion tensor imaging (DTI) axial volumes were obtained using a multishell diffusion weighting strategy along 107 independent directions with b-values encapsulating shells at 300, 700, 1000, 2000, and 3000 s/mm^2^ for comprehensive diffusion characterization. Preprocessing steps included denoising, eddy current, and head motion correction involving MRtrix3 and FSL software packages. Cortical reconstruction and segmentation were performed with the FreeSurfer software package. After co-registration of individual anatomical and diffusion spaces, a 5-tissue type segmentation based on Hybrid Surface and Volume Segmentation was performed for the use of Anatomically Constrained Tractography. A 10 million streamlines whole-brain tractogram was then generated through iFOD2 probabilistic algorithm using a cut-off of 0.1 and backtrack propagation. Fiber assignment was done with a radial search of 2mm. The connectome matrix was generated after filtering streamlines using the SIFT2 method. As for the parcellation, we used the 4th homotopic Schaefer atlas of 200 regions of interest (ROIs) [91].

Source-reconstructed signals were localized on the same ROIs using a weight minimum norm estimation (wMNE) with a regularization parameter set to 1/3^2^ based on the forward solution Boundary Element Method (BEM) computed on the individual MRI of the stroke patient [92]. For each ROI, we estimated the power spectrum (PSD) using the Welch method with a windowing of 1.5 s, an overlap of 0.25 s, and a 1 Hz frequency resolution, i.e. 0, 1, …, 40 Hz. Then, we considered the values extracted at 10 Hz and we averaged consecutive trials by blocks of six so to have more robust estimation. Eventually, we obtained 10 blocks per condition that were used for the subsequent network controllability analysis.

## Supporting information

S1 Text Optimal control: input signal derivation, Laplacian of the network and state matrix. (DOCX)

S1 FigEffects of the parameters and topology.a) Precision *δ* as a function of the simulation parameters. The final states *x*_*f*_ are sampled from a Gaussian distribution with a fixed mean of *μ*_*f*_ = 1 and standard deviation *σ*_*f*_ *=* 10. Number of drivers *n*_*d*_ *=* 1 and number of eigenmaps *r* = 4, 16, 64, or 256. Left panel = reciprocal of the regularization parameter 1/*ρ*. Right panel = the final time *t*_*f*_. when controlling eigenmaps. The parameters are chosen within the ranges corresponding to good performance in terms of precision, i.e., *ρ* = 10^−4^, *dτ =* 0.01 and *t*_*f*_ = 1. Results are averaged over 100 HMSW network realizations. The inset shows that the lower the parameter *ρ*, the higher the energy *E* of the control signal for steering the system. b) Control accuracy in terms of precision *δ* and representativeness *η* as a function of the dispersion (i.e., standard deviation *σ*_*f*_) of the network nodes’ states. The final states *x*_*f*_ are sampled from a Gaussian distribution with a fixed mean of *μ*_*f*_ *=* 1. Number of drivers *n*_*d*_ *=* 1 and number of eigenmaps *r* = 4, 16, 64, or 256. Results are averaged over 100 HMSW network realizations. c) Representativeness *η* and precision *δ* (inset) as a function of the final state dispersion *σ*_*f*_ using *r* = 64 most representative eigenmaps sorted according to the magnitude of the eigenstate (in blue) or using the *r* first eigenmaps corresponding to the smaller Laplacian eigenvalues (in red). We used all nodes as drivers *n*_*d*_ = *n* and the final state had a fixed mean of *μ*_*f*_ = 1. for smooth final states with small dispersion *σ*_*f*_, both eigenmaps selection schemes give high and similar representativeness *η* with a slight superiority of the *λ*-sorted scheme. As the dispersion increased, *σ*_*f*_>1, selecting the eigenmaps accordingly to the eigenstate gives better representativeness *η* and that is accompanied by lower precision *δ* in the low-dimensional space as the control task gets harder. Results are averaged over 100 HMSW network realizations and shaded areas represent standard deviations. d) Precision *δ*, representativeness *η*, and their sum as a function of the number of controlled eigenmaps *r* for two different topologies: the Erdos-Renyi model ER (*n* = 256, rewiring probability *p* = 0.035), and the Barabasi-Albert model BA (*n* = 256, connection density 0.035, bias *γ* = 2). The control task is the same as in c) with the final state sampled from $N(\mu_{f}=1,\;{\;\sigma }_{f}=10)$, and the trajectory simulated for *ρ* = 10^−4^ and *t*_*f*_ = 1. Results are averaged over 100 network realizations.(DOCX)

S2 Fig Effect of time resolution *dτ* on control precision. a) According to the Forward Euler discretization/integration method, the theoretical condition for stability is that *dt <* 2 */max*(|*Re(λ*_*i*_*)|*) with the eigenvalues calculated from the connectivity matrix A. The histogram shows the values obtained from 100 networks generated with the HMSW model. The solid vertical line indicates the chosen value *dτ =* 0.01. b) PCA projection of the output state trajectory for different time resolution values. Data from one generated HMSW network are shown here for illustrative purposes. The input control signal is obtained by solving Eq 3 with same parameters as in Fig 2b, with *n*_*d*_ *=* 8 and *r* = 8. c) Control precision as function of the number of eigenmaps. Different markers correspond to different time resolutions. Different colors indicate a different number of drivers. Values are averaged across simulations obtained from 100 HMSW networks. The input control signals are obtained by solving Eq 3 with same parameters as in Fig 2b.
(DOCX)

S3 FigControl accuracy for different configurations of the network nodes’ states.a) Uniform-Modular network states’ distributions. Left panel: the structural network is generated with the same hierarchical modular small-world model (HSWM) used in Fig 1. The distribution of the nodes’ states for the initial state *x*_0_ is sampled from a continuous uniform distribution *U(-1*,*1)*. For the final state *x*_*f*_ we grouped the nodes in eight modules based on the structure inherited by the HSWM and assigned their states sampling from the distribution *i+U(-1*,*1)*, where *i = 1*,*2*,..*8*. The color of the nodes indicates how values are distributed in the network. One-hundred networks are generated. In average, the resulting initial state has a mean *μ*_0_ = 0 and standard deviation *σ*_0_ = 0.58; for the final state *μ*_*f*_ = 4.5 and *σ*_*f*_ *=* 2.4. The middle and right panel show respectively the average control precision and representativeness as a function of the number of eigenmaps (*r*). Different colored lines indicate different number of driver nodes, selected based on their highest between centrality. The simulation parameters to solve Eq 1 are *t*_*f*_ *=* 1 and *ρ* = 0.0083, 0.0113, 0.0234 respectively for one, eight and 64 drivers. b) Constant-Gaussian network states’ distributions. Left panel: the values of the nodes’ states are also sampled from a continuous uniform distribution *U(-1*,*1)*. For the final state *x*_*f*_ values are sampled from a Gaussian distribution *$N\left(\mu_{f}=4.5,\;\sigma_{f}=2.4\right)$*. The middle and right panel show respectively the average control precision and representativeness as a function of the number of eigenmaps (*r*). Same parameters and graphical conventions as in a). Although, the values in the Uniform-Modular configuration are slightly higher than those in the Constant-Gaussian configuration, the global trend stays the same as a function of the number of eigenmaps.(DOCX)

S4 Fig Effect of network size (n) on different control performance metrics. Values are averaged across simulations obtained from 100 HMSW networks. The input control signals are obtained by solving Eq 3 with same parameters as in Fig 2b using *n*_*d*_ *=* 8 and ρ = 10^−1^. Different color lines correspond to different numbers of eigenmaps. a) Control precision (*δ*) b) Control energy (*E*).
(DOCX)

S5 FigControl precision as a function of the time horizon for the experimental brain dataset (Materials and Methods).In brain networks, communication dynamics are constrained by the axonal conduction delay between directly connected regions. To account for this, we rescaled the connectivity matrix A so that its minimum eigenvalue -representing the fastest communication mode *λ*_fast_- matches the typical interareal conduction delay $\tilde{\tau }=0.0102$ seconds. It is straightforward to demonstrate that, under these conditions, the rescaling formula becomes $\tilde{A}=-\frac{1}{\tilde{\tau }\lambda_{fast}}A$. The control signal from one driver is obtained using $\tilde{A}$ and keeping the other parameters as in Fig 3d. The shaded area highlights the tf values for which the precision stays relatively high regardless of the number of selected eigenmaps r. This corresponds to an interval between 0.1 and 0.3 seconds. Precision becomes extremely poor for time horizons longer than 2 seconds. Solid lines show the values averaged over 10 experimental blocks. Vertical bars indicate standard deviations.(DOCX)

S6 FigSingle-driver controllability of target brain networks: effect of dimension.Low-dimensional worst-case control centrality $\lambda_{min}^{EIG}$ values as a function of the number of eigenmaps *r*. For each of the 9 columns and colors, a different brain system is taken as target and each point corresponds to a different node (ROI). Values are shown for a representative subject. By decreasing *r*, all $\lambda_{min}^{EIG}$ values become positive and numerically reliable after a critical threshold r*. The inset illustrates the distribution of r* from all subjects (N = 6134). The group median ${\tilde{r}}^{*}=5$ has been chosen as representative value for each subject. Note that the $\lambda_{min}^{EIG}$ is equivalent to the standard metric *λ*_*min*_ when *r = m*.(DOCX)

S7 FigRelationship between low-dimensional controllability and distance metrics.a) Pearson correlation between low-dimensional controllability and distances between drivers and targeted networks. We considered the distance to the targeted network as the sum of the distances to its nodes $d_{i,\;S_{net}}=\sum_{j\in S_{net}}d_{i,j}$. Topological (blue) refers to the length of the shortest path and spatial (red) to the Euclidean distance. b) Visualization of the correlation coefficients and scatter plots for two representative brain systems, i.e. the VIS network regarding topological distance and the DAN regarding spatial distance.(DOCX)

S8 FigControl relationships between brain systems in a finer parcellation.a) The 17 split components of the Yeo2011 brain atlas parcellation [36]. b) Adjacency matrix of the group-averaged controllability meta-graph between the different brain systems. The links represented by color are the group-average of control centrality of the system in column *i* when targeting system in line *j ${\langle \lambda_{min}^{EIG}\rangle }_{i\to j}$*. ${\langle \lambda_{min}^{EIG}\rangle }_{i\to j}$ is obtained by taking the geometric mean control centrality $\lambda_{min}^{EIG}$ of drivers in system *i* when targeting system in *j*. Self-loops and the SUB network are not represented as their control centrality is several orders of magnitude higher. c) System total contribution as the sum of outgoing and incoming weighted links from the individual meta-graphs. Bars indicate group-average values and error bars standard deviations. d) System control unbalance as the difference between the sum of outgoing and incoming weighted links from the individual meta-graphs. Positive values = tendency to act as driver. Negative value = tendency to act as target. Bars indicate group-average values and error bars standard deviations.(DOCX)

S9 FigRelationship between low-dimensional controllability and node strength.a) Group-averaged spatial distribution of node strength k given by the sum of all the weighted links of a node. b) Group-averaged spatial distribution of low-dimensional ($\lambda_{min}^{EIG}$) control centrality. c) Scatter plot of node strength k and low-dimensional ($\lambda_{min}^{EIG}$) control centrality for all *n* × *N* nodes and participants. Pearson correlation test revealed a low correlation between the two metrics.(DOCX)

S1 File Labels of the regions of interest (ROIs) used in the parcellation of the Schaefer atlas. (XLSX)

S2 File Details of performed statistical tests. (XLSX)
