# Correction: Low-dimensional controllability of brain networks

**DOI:** 10.1371/journal.pcbi.1013601

**Published:** 2025-10-21

**Authors:** Remy Ben Messaoud, Vincent Le Du, Camile Bousfiha, Marie-Constance Corsi, Juliana Gonzalez-Astudillo, Brigitte Charlotte Kaufmann, Tristan Venot, Baptiste Couvy-Duchesne, Raffaella Migliaccio, Charlotte Rosso, Paolo Bartolomeo, Mario Chavez, Fabrizio De Vico Fallani

## Notice of republication

This article was republished on September 25, 2025, to correct errors the ninth author’s name. The correct name is: Raffaella Migliaccio. The originally published, uncorrected article and the republished, corrected articles are provided here for reference.

## Supporting information

S1 File Originally published, uncorrected article. (PDF)

S2 File Republished, corrected article. (PDF)
