# Supplementary material for: Low-dimensional controllability of brain networks
Source: PLoS Comput Biol. 2025 Jan 7;21(1):e1012691. doi: 10.1371/journal.pcbi.1012691 (PMC11706394; doi:10.1371/journal.pcbi.1012691)
Supplement: S1 Text — (DOCX) [file pcbi.1012691.s001.docx]

**S1 Text**

**Optimal control: input signal derivation**

In the results section, we validated our framework for synthetic networks by simulating trajectories. We first, need to find the control input $u(t)$. To do so we solve the following optimization problem:

|  | $\begin{aligned} {min}_{u} \{ J_{\rho}(u, t_{f})=\left( y_{f} -y\left( t_{f} \right) \right)^{T}\left( y_{f} -y\left( t_{f} \right) \right)+\rho\int_{0}^{t_{f}} {u\left( \tau\right)}^{T}u\left( \tau\right) d\tau\} \\ s.t. \dot{x}\left( t \right)=Ax\left( t \right)+Bu\left( t \right); y\left( t \right)=Cx\left( t \right); x\left( 0 \right)=x_{0} \end{aligned}$ | (1) |
| --- | --- | --- |

Overall, it is a minimization problem with *soft constraints* on the output. The term $\rho\int_{0}^{t_{f}} {u\left( \tau\right)}^{T}u\left( \tau\right)d\tau$, called the scalar running cost function, minimizes the energy, and the term

$\left( y_{f} -y\left( t_{f} \right) \right)^{T}\left( y_{f} -y\left( t_{f} \right) \right)=\left( y_{f} -Cx\left( t_{f} \right) \right)^{T}\left( y_{f} -Cx\left( t_{f} \right) \right)$, called the scalar terminal cost function, is where we express the final soft constraint on the output.

The problem can be solved using Pontriyargin’s maximum principle by introducing the Hamiltonian equation:

|  | $\mathcal{H}\left( x\left( t \right),v\left( t \right),u\left( t \right) \right)= \rho{u\left( t \right)}^{T}u\left( t \right)+ {v\left( t \right)}^{T}\left( Ax\left( t \right)+Bu(t) \right)$, | (2) |
| --- | --- | --- |

where $v\left( t \right)$ is the vector of the adjoint states.

We know from the fundamentals of optimal control theory^2^ that the optimal trajectory $(x^{*},v^{*},u^{*})$ is the solution to the following equations:

|  | - State equation: $\frac{\partial\mathcal{H}}{\partial v}=\dot{x}\left( t \right)=Ax\left( t \right)+Bu(t)$ | (3) |
| --- | --- | --- |
|  | - Adjoint equation: $-\frac{\partial\mathcal{H}}{\partial x}=\dot{v}\left( t \right)=-A^{T}v(t)$ | (4) |
|  | - Stationary equation: $0=\frac{\partial\mathcal{H}}{\partial u}=2\rho u\left( t \right)+B^{T}v(t)$ | (5) |
|  | - Boundary/Transversality condition: $v\left( t_{f} \right)=\frac{\partial E(x(t_{f}))}{\partial x(t_{f})}=-2C^{T}( y_{f} -Cx\left( t_{f} \right))$ | (6) |

The adjoint and stationary equations, (4) and (5), can be rewritten in condensed form:

|  | $\left[ \begin{matrix} \dot{x^{*}} \\ \dot{v^{*}} \end{matrix} \right]=\left[ \begin{matrix} A & -{(2\rho)}^{-1}BB^{T} \\ 0 & -A^{T} \end{matrix} \right] \left[ \begin{matrix} x^{*} \\ v^{*} \end{matrix} \right]=H \left[ \begin{matrix} x^{*} \\ v^{*} \end{matrix} \right]$ | (7) |
| --- | --- | --- |

where $H$ is the Hamiltonian matrix. Equation (7) can be solved as:

|  | $\left[ \begin{matrix} x^{*}(t) \\ v^{*}(t) \end{matrix} \right]=e^{tH}\left[ \begin{matrix} x^{*}(0) \\ v^{*}(0) \end{matrix} \right]$ | (8) |
| --- | --- | --- |

This way, we obtained an expression for $v^{*}(t)$ and the problem is almost solved since equation (5) gives $u^{*}\left( t \right)=\frac{-B^{T}v(t)}{2\rho}$. We still are left with the unknown $v^{*}(0)$, which can be found using the boundary conditions^3^. If we note $e^{t_{f} H}=\left[ \begin{matrix} M_{11} & M_{12} \\ M_{21} & M_{22} \end{matrix} \right]$, we have the three following boundary constraints:

|  | $\left\{ \begin{aligned} x^{*}\left( t_{f} \right)=M_{11}x_{0}+M_{12}v^{*}\left( 0 \right) \\ v^{*}\left( t_{f} \right)=M_{21}x_{0}+M_{22}v^{*}\left( 0 \right) \\ v^{*}\left( t_{f} \right)=-{2C}^{T}( y_{f} -Cx^{*}\left( t_{f} \right)) \end{aligned} \right.$ | (9) |
| --- | --- | --- |

The first two equations come from (8) and the third is the Hamiltonian boundary condition (6). We have three unknowns $v^{*}\left( 0 \right)$, $x^{*}\left( t_{f} \right)$, $v^{*}\left( t_{f} \right)$, and three equations. The calculus gives:

|  | $v^{*}\left( 0 \right)=\left( M_{22}-{2C}^{T}CM_{12} \right)^{\dagger}(({2C}^{T}CM_{11}-M_{21})x_{0}-{2C}^{T}y_{f})$, | (10) |
| --- | --- | --- |

where $\dagger$ denotes the pseudo-inverse of a matrix.

**Laplacian of the network and state matrix**

For a graph with adjacency matrix $G$ the Laplacian matrix is defined as $L\left( G \right)= D_{G}-G$, where $D_{G}$ is the diagonal matrix containing the degree sequence of nodes of $G$. In this work, we stabilize the nodes’ dynamics and consider the matrix $A=G-cI$, where $c$ is an arbitrary constant. We verify here, using the linearity of the Laplacian operator, that $L\left( A \right)=L\left( G \right)$.

|  | $L\left( A \right)=L\left( G-cI \right)=L\left( G \right)-L\left( cI \right)=\left( D_{G}-G \right)-\left( cI-cI \right)=D_{G}-G=L(G)$ | (11) |
| --- | --- | --- |
