# Supplementary material for: Low-dimensional controllability of brain networks
Source: PLoS Comput Biol. 2025 Jan 7;21(1):e1012691. doi: 10.1371/journal.pcbi.1012691 (PMC11706394; doi:10.1371/journal.pcbi.1012691)
Supplement: S1 Fig — a) Precision δ as a function of the simulation parameters. The final states xf are sampled from a Gaussian distribution with a fixed mean of μf = 1 and standard deviation σf = 10. Number of drivers nd = 1 and number of eigenmaps r = 4, 16, 64, or 256. Left panel = reciprocal of the regularization parameter 1/ρ. Right panel = the final time tf. when controlling eigenmaps. The parameters are chosen within the ranges corresponding to good performance in terms of precision, i.e., ρ = 10−4, dτ = 0.01 and tf = 1. Results are averaged over 100 HMSW network realizations. The inset shows that the lower the parameter ρ, the higher the energy E of the control signal for steering the system. b) Control accuracy in terms of precision δ and representativeness η as a function of the dispersion (i.e., standard deviation σf) of the network nodes’ states. The final states xf are sampled from a Gaussian distribution with a fixed mean of μf = 1. Number of drivers nd = 1 and number of eigenmaps r = 4, 16, 64, or 256. Results are averaged over 100 HMSW network realizations. c) Representativeness η and precision δ (inset) as a function of the final state dispersion σf using r = 64 most representative eigenmaps sorted according to the magnitude of the eigenstate (in blue) or using the r first eigenmaps corresponding to the smaller Laplacian eigenvalues (in red). We used all nodes as drivers nd = n and the final state had a fixed mean of μf = 1. for smooth final states with small dispersion σf, both eigenmaps selection schemes give high and similar representativeness η with a slight superiority of the λ-sorted scheme. As the dispersion increased, σf>1, selecting the eigenmaps accordingly to the eigenstate gives better representativeness η and that is accompanied by lower precision δ in the low-dimensional space as the control task gets harder. Results are averaged over 100 HMSW network realizations and shaded areas represent standard deviations. d) Precision δ, representativeness η, and the [file pcbi.1012691.s002.docx]

**
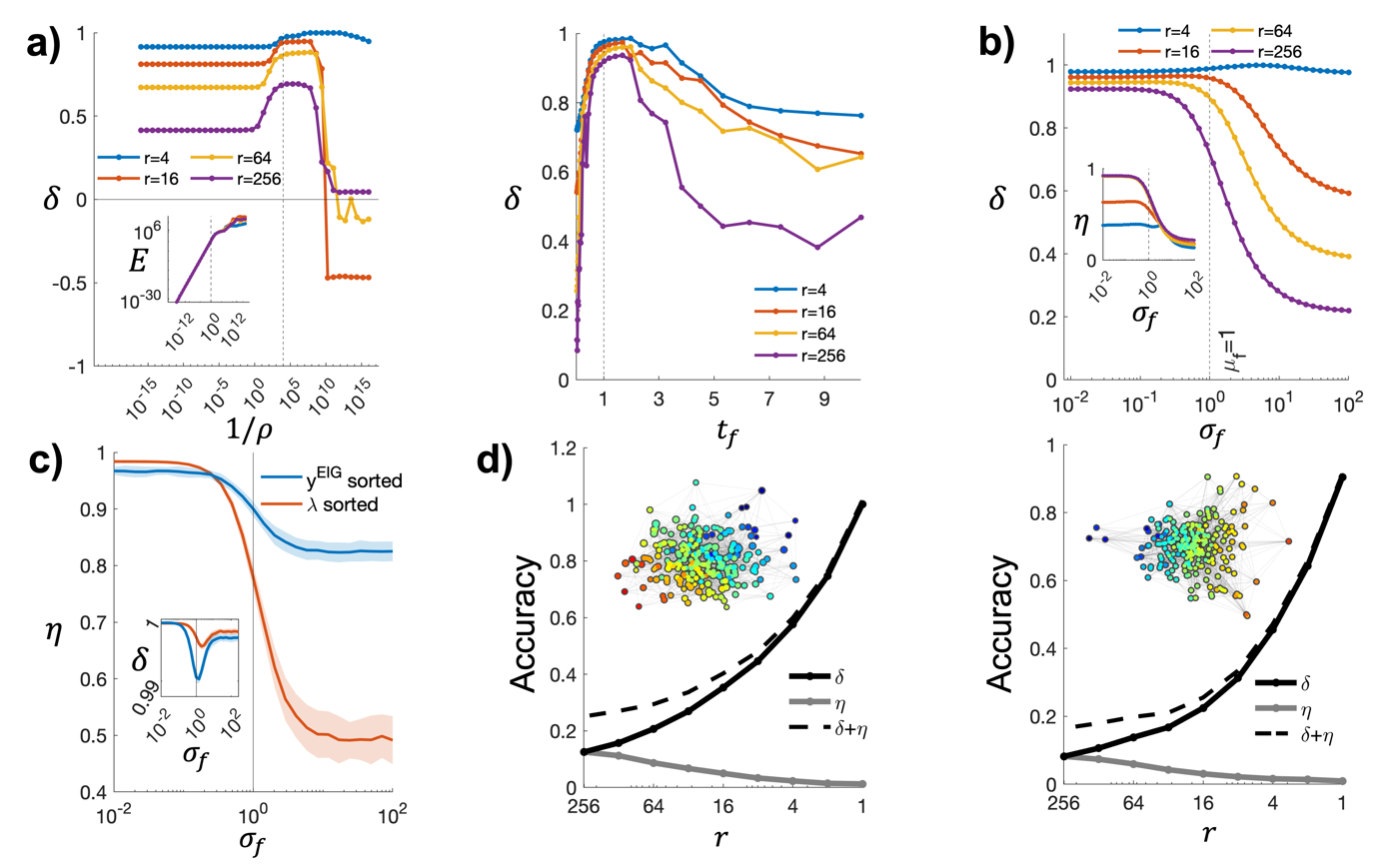
**

**S1 Fig. Effects of the parameters and topology**

1. Precision $\delta$ as a function of the simulation parameters. The final states $x_{f}$ were sampled from a Gaussian distribution with a fixed mean of $\mu_{f}$=1 and standard deviation $\sigma_{f}=10$. Number of drivers $n_{d}=1$ and number of eigenmaps $r$ = 4, 16, 64, or 256. Left panel=reciprocal of the regularization parameter $1/\rho$. Right panel=the final time $t_{f}$. when controlling eigenmaps. The parameters are chosen within the ranges corresponding to good performance in terms of precision, i.e., $\rho$ =10^-4 ,^ $d\tau=0.01$ and $t_{f}$ = 1. Results are averaged over 100 HMSW network realizations. The inset shows that the lower the parameter $\rho$, the higher the energy *E* of the control signal for steering the system.
2. Control accuracy in terms of precision $\delta$ and representativeness $\eta$ as a function of the dispersion (i.e., standard deviation $\sigma_{f})$ of the network nodes’ states. The final states $x_{f}$ were sampled from a Gaussian distribution with a fixed mean of $\mu_{f}$= 1. Number of drivers $n_{d}=1$ and number of eigenmaps $r$ = 4, 16, 64, or 256. Results are averaged over 100 HMSW network realizations.
3. Representativeness $\eta$ and precision $\delta$ (inset) as a function of the final state dispersion $\sigma_{f}$ using $r$=64 most representative eigenmaps sorted according to the magnitude of the eigenstate (in blue) or using the $r$ first eigenmaps corresponding to the smaller Laplacian eigenvalues (in red). We used all nodes as drivers $n_{d}$=$n$ and the final state had a fixed mean of $\mu_{f}$=1. for smooth final states with small dispersion $\sigma_{f}$, both eigenmaps selection schemes gave high and similar representativeness $\eta$ with a slight superiority of the $\lambda$-sorted scheme. As the dispersion increased, $\sigma_{f}$>1, selecting the eigenmaps accordingly to the eigenstate gave better representativeness $\eta$ and that was accompanied by lower precision $\delta$ in the low-dimensional space as the control task was harder. Results are averaged over 100 HMSW network realizations and shaded areas represent standard deviations.

Precision $\delta$, representativeness $\eta$, and their sum as a function of the number of controlled eigenmaps $r$ for two different topologies: the Erdos-Renyi model ER ($n$=256, $p$=0.035), and the Barabasi-Albert model BA ($n$=256 and connection density 0.035, bias $\gamma$= 2). d de. We also the same control task as for the HMSW model: the final state was sampled from $\mathcal{N(}\mu_{f}\text{=1,} \sigma_{f}\text{=10)}$, and the trajectory was simulated for $\rho$=10^-4^ and $t_{f}$=1. Results are averaged over 100 HMSW network realizations.
