# Supplementary material for: Low-dimensional controllability of brain networks
Source: PLoS Comput Biol. 2025 Jan 7;21(1):e1012691. doi: 10.1371/journal.pcbi.1012691 (PMC11706394; doi:10.1371/journal.pcbi.1012691)
Supplement: S2 Fig — a) According to the Forward Euler discretization/integration method, the theoretical condition for stability is that dt < 2 /max(|Re(λi)|) with the eigenvalues calculated from the connectivity matrix A. The histogram shows the values obtained from 100 networks generated with the HMSW model. The solid vertical line indicates the chosen value dτ = 0.01. b) PCA projection of the output state trajectory for different time resolution values. Data from one generated HMSW network are shown here for illustrative purposes. The input control signal is obtained by solving Eq 3 with same parameters as in Fig 2b, with nd = 8 and r = 8. c) Control precision as function of the number of eigenmaps. Different markers correspond to different time resolutions. Different colors indicate a different number of drivers. Values are averaged across simulations obtained from 100 HMSW networks. The input control signals are obtained by solving Eq 3 with same parameters as in Fig 2b. (DOCX) [file pcbi.1012691.s003.docx]

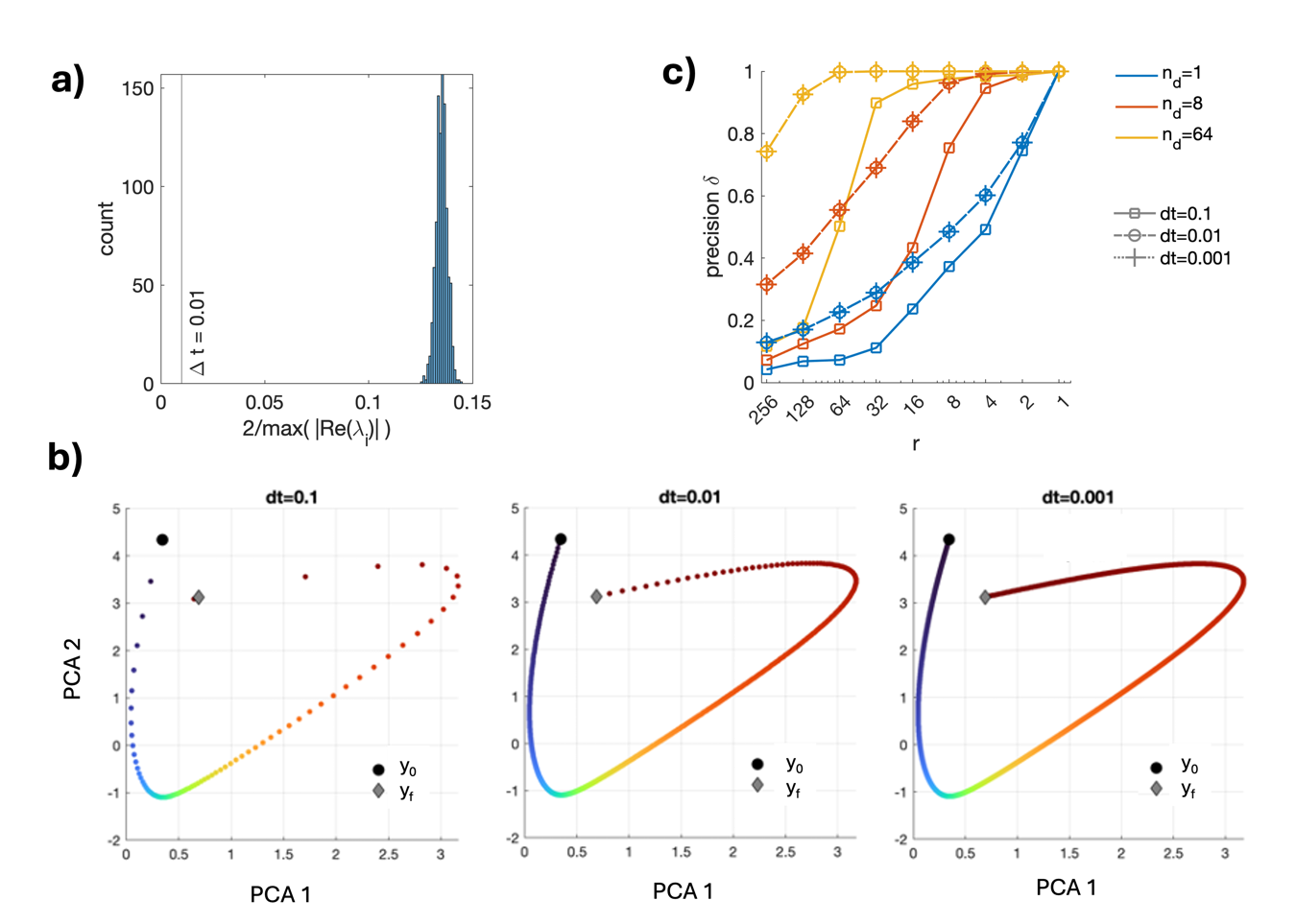


**S2 Fig.** **Effect of time resolution** $d\tau$ **on control precision.**

1. According to the Forward Euler discretization/integration method, the theoretical condition for stability is that $dt<2/max(|Re(\lambda_{i})|)$ with the eigenvalues calculated from the connectivity matrix A. The histogram shows the values obtained from 100 networks generated with the HMSW model. The solid vertical line indicates the chosen value $d\tau=0.01$.
2. PCA projection of the output state trajectory for different time resolution values. Data from one generated HMSW network are shown here for illustrative purposes. The input control signal is obtained by solving Eq. 3 with same parameters as in **Fig 2b**, with $n_{d}=8$ and $r=8$.
3. Control precision as function of the number of eigenmaps. Different markers correspond to different time resolutions. Different colors indicate a different number of drivers. Values are averaged across simulations obtained from 100 HMSW networks. The input control signals are obtained by solving Eq. 3 with same parameters as in **Fig 2b.**
