# Supplementary material for: Low-dimensional controllability of brain networks
Source: PLoS Comput Biol. 2025 Jan 7;21(1):e1012691. doi: 10.1371/journal.pcbi.1012691 (PMC11706394; doi:10.1371/journal.pcbi.1012691)
Supplement: S3 Fig — a) Uniform-Modular network states’ distributions. Left panel: the structural network is generated with the same hierarchical modular small-world model (HSWM) used in Fig 1. The distribution of the nodes’ states for the initial state x0 is sampled from a continuous uniform distribution U(-1,1). For the final state xf we grouped the nodes in eight modules based on the structure inherited by the HSWM and assigned their states sampling from the distribution i+U(-1,1), where i = 1,2,..8. The color of the nodes indicates how values are distributed in the network. One-hundred networks are generated. In average, the resulting initial state has a mean μ0 = 0 and standard deviation σ0 = 0.58; for the final state μf = 4.5 and σf = 2.4. The middle and right panel show respectively the average control precision and representativeness as a function of the number of eigenmaps (r). Different colored lines indicate different number of driver nodes, selected based on their highest between centrality. The simulation parameters to solve Eq 1 are tf = 1 and ρ = 0.0083, 0.0113, 0.0234 respectively for one, eight and 64 drivers. b) Constant-Gaussian network states’ distributions. Left panel: the values of the nodes’ states are also sampled from a continuous uniform distribution U(-1,1). For the final state xf values are sampled from a Gaussian distribution Nμf=4.5,σf=2.4. The middle and right panel show respectively the average control precision and representativeness as a function of the number of eigenmaps (r). Same parameters and graphical conventions as in a). Although, the values in the Uniform-Modular configuration are slightly higher than those in the Constant-Gaussian configuration, the global trend stays the same as a function of the number of eigenmaps. (DOCX) [file pcbi.1012691.s004.docx]

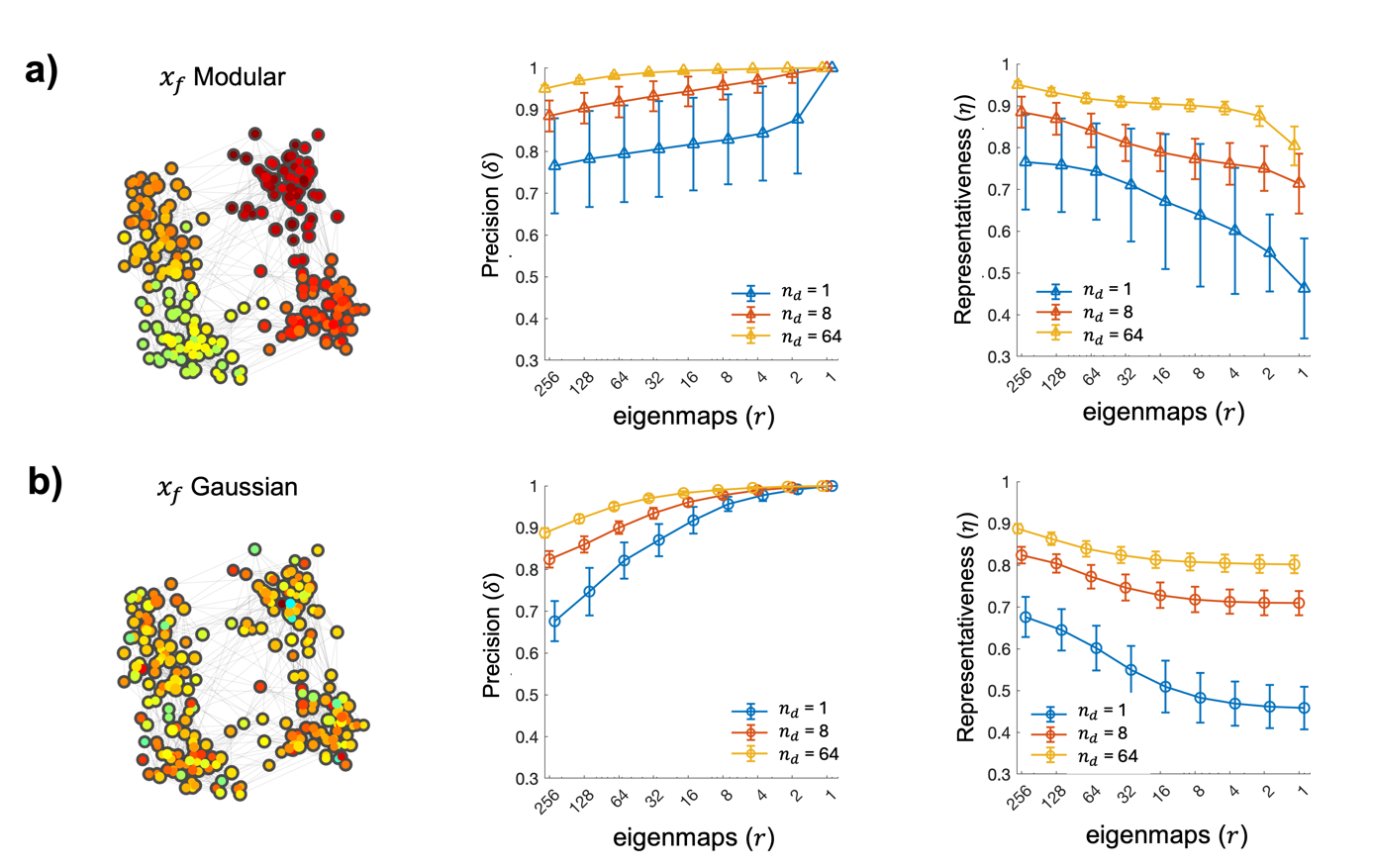


**S3 Fig. Control accuracy for different configurations of the network nodes’ states.**

1. Uniform-Modular network states’ distributions. Left panel: the structural network is generated with the same hierarchical modular small-world model (HSWM) used in Fig 1. The distribution of the nodes’ states for the initial state $x_{0}$ is sampled from a continuous uniform distribution *U(-1,1).* For the final state $x_{f}$ we grouped the nodes in eight modules based on the structure inherited by the HSWM and assigned their states sampling from the distribution *i+U(-1,1)*, where *i=1,2,..8.* The color of the nodes indicates how values are distributed in the network. One-hundred networks are generated. In average, the resulting initial state has a mean $\mu_{0}=0$ and standard deviation $\sigma_{0}=0.58$; for the final state $\mu_{f}=4.5$ and $\sigma_{f}=2.4$.

The middle and right panel show respectively the average control precision and representativeness as a function of the number of eigenmaps ($r$). Different colored lines indicate different number of driver nodes, selected based on their highest between centrality. The simulation parameters to solve Eq. 1 are $t_{f}=1$ and $\rho=$ 0.0083, 0.0113, 0.0234 respectively for one, eight and 64 drivers.

Constant-Gaussian network states’ distributions. Left panel: the values of the nodes’ states are also sampled from a continuous uniform distribution *U(-1,1)*. For the final state $x_{f}$ values are sampled from a Gaussian distribution $\mathcal{N}\left( \mu_{f}=4.5 ,\sigma_{f}=2.4 \right)$. The middle and right panel show respectively the average control precision and representativeness as a function of the number of eigenmaps ($r$). Same parameters and graphical conventions as in a). Although, the values in the Uniform-Modular configuration are slightly higher than those in the Constant-Gaussian configuration, the global trend stays the same as a function of the number of eigenmaps.
