# Supplementary material for: Low-dimensional controllability of brain networks
Source: PLoS Comput Biol. 2025 Jan 7;21(1):e1012691. doi: 10.1371/journal.pcbi.1012691 (PMC11706394; doi:10.1371/journal.pcbi.1012691)
Supplement: S4 Fig — Values are averaged across simulations obtained from 100 HMSW networks. The input control signals are obtained by solving Eq 3 with same parameters as in Fig 2b using nd = 8 and ρ = 10−1. Different color lines correspond to different numbers of eigenmaps. a) Control precision (δ) b) Control energy (E). (DOCX) [file pcbi.1012691.s005.docx]

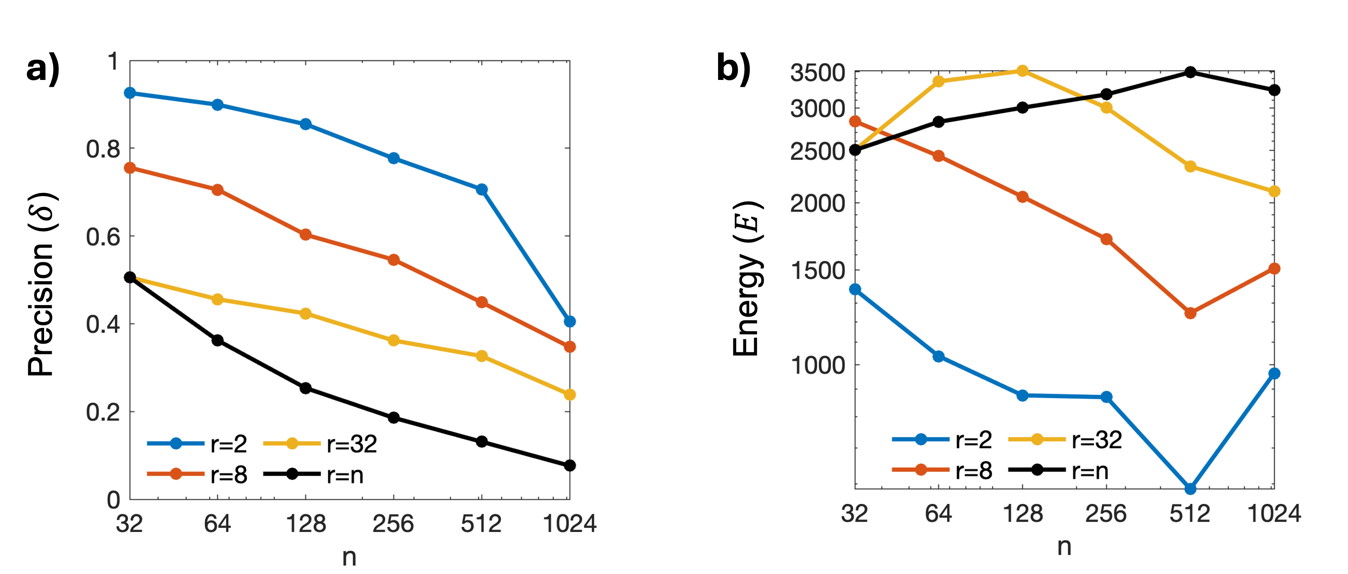


**S4 Fig.** – **Effect of network size (n) on different control performance metrics.**

Values are averaged across simulations obtained from 100 HMSW networks. The input control signals are obtained by solving Eq. 3 with same parameters as in **Fig 2b** using $n_{d}=8$ and $\rho={10}^{-1}$. Different color lines correspond to different numbers of eigenmaps.

1. Control precision ($\delta$)
2. Control energy (*E*)
