# Supplementary material for: Low-dimensional controllability of brain networks
Source: PLoS Comput Biol. 2025 Jan 7;21(1):e1012691. doi: 10.1371/journal.pcbi.1012691 (PMC11706394; doi:10.1371/journal.pcbi.1012691)
Supplement: S5 Fig — In brain networks, communication dynamics are constrained by the axonal conduction delay between directly connected regions. To account for this, we rescaled the connectivity matrix A so that its minimum eigenvalue -representing the fastest communication mode λfast- matches the typical interareal conduction delay τ~=0.0102 seconds. It is straightforward to demonstrate that, under these conditions, the rescaling formula becomes A~=-1τ~λfastA. The control signal from one driver is obtained using A~ and keeping the other parameters as in Fig 3d. The shaded area highlights the tf values for which the precision stays relatively high regardless of the number of selected eigenmaps r. This corresponds to an interval between 0.1 and 0.3 seconds. Precision becomes extremely poor for time horizons longer than 2 seconds. Solid lines show the values averaged over 10 experimental blocks. Vertical bars indicate standard deviations. (DOCX) [file pcbi.1012691.s006.docx]

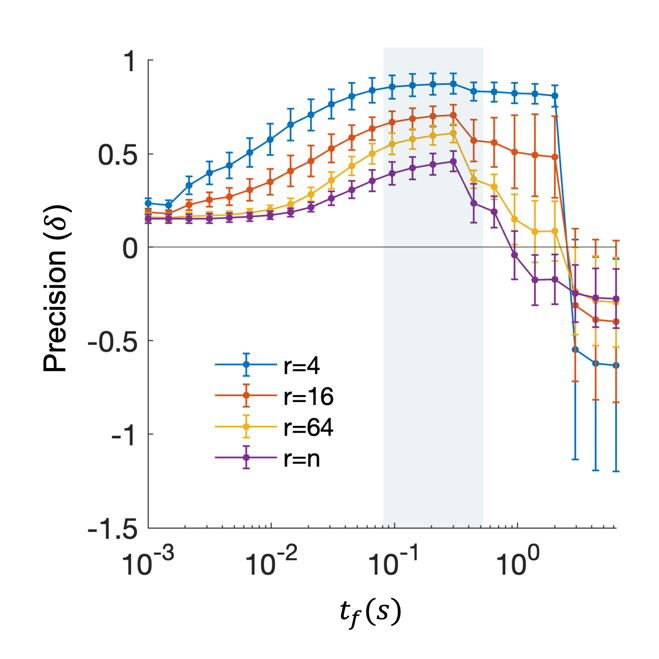


| **S5 Fig** - **Control precision as a function of the time horizon for the experimental brain dataset** (**Materials and Methods**).  In brain networks, communication dynamics are constrained by the axonal conduction delay between directly connected regions. To account for this, we rescaled the connectivity matrix A so that its minimum eigenvalue -representing the fastest communication mode $\lambda_{fast}$- matches the typical interareal conduction delay $\tilde{\tau}=0.0102$ seconds. It is straightforward to demonstrate that, under these conditions, the rescaling formula becomes $\tilde{A}=-\frac{1}{\tilde{\tau}\lambda_{fast}}A$.  The control signal from one driver is obtained using $\tilde{A}$ and keeping the other parameters as in **Fig 3d.** The shaded area highlights the $t_{f}$ values for which the precision stays relatively high regardless of the number of selected eigenmaps $r$. This corresponds to an interval between 0.1 and 0.3 seconds. Precision becomes extremely poor for time horizons longer than 2 seconds. Solid lines show the values averaged over 10 experimental blocks. Vertical bars indicate standard deviations. |
| --- |
