# Supplementary material for: Low-dimensional controllability of brain networks
Source: PLoS Comput Biol. 2025 Jan 7;21(1):e1012691. doi: 10.1371/journal.pcbi.1012691 (PMC11706394; doi:10.1371/journal.pcbi.1012691)
Supplement: S6 Fig — Low-dimensional worst-case control centrality λminEIG values as a function of the number of eigenmaps r. For each of the 9 columns and colors, a different brain system is taken as target and each point corresponds to a different node (ROI). Values are shown for a representative subject. By decreasing r, all λminEIG values become positive and numerically reliable after a critical threshold r*. The inset illustrates the distribution of r* from all subjects (N = 6134). The group median r~*=5 has been chosen as representative value for each subject. Note that the λminEIG is equivalent to the standard metric λmin when r = m. (DOCX) [file pcbi.1012691.s007.docx]

**S6 Fig. Single-driver controllability of target brain networks: effect of dimension.**

Low-dimensional worst-case control centrality $\lambda_{min}^{EIG}$ values as a function of the number of eigenmaps $r$. For each of the 9 columns and colors, a different brain system is taken as target and each point corresponds to a different node (ROI). Values are shown for a representative subject. By decreasing $r$, all $\lambda_{min}^{EIG}$ values become positive and numerically reliable after a critical threshold $r^{*}$. The inset illustrates the distribution of $r^{*}$from all subjects (N=6134). The group median $\tilde{r}^{*}=5$ has been chosen as representative value for each subject. Note that the $\lambda_{min}^{EIG}$ is equivalent to the standard metric $\lambda_{min}$ when $r=m$.
