# Supplementary material for: Low-dimensional controllability of brain networks
Source: PLoS Comput Biol. 2025 Jan 7;21(1):e1012691. doi: 10.1371/journal.pcbi.1012691 (PMC11706394; doi:10.1371/journal.pcbi.1012691)
Supplement: S7 Fig — a) Pearson correlation between low-dimensional controllability and distances between drivers and targeted networks. We considered the distance to the targeted network as the sum of the distances to its nodes di,Snet=∑j∈Snetdi,j. Topological (blue) refers to the length of the shortest path and spatial (red) to the Euclidean distance. b) Visualization of the correlation coefficients and scatter plots for two representative brain systems, i.e. the VIS network regarding topological distance and the DAN regarding spatial distance. (DOCX) [file pcbi.1012691.s008.docx]

**S7 Fig. Relationship between low-dimensional controllability and distance metrics.**

1. Pearson correlation between low-dimensional controllability and distances between drivers and targeted networks. We considered the distance to the targeted network as the sum of the distances to its nodes $d_{i, S_{net}}=\sum_{j\in S_{net}} d_{i,j}$. Topological (blue) refers to the length of the shortest path and spatial (red) to the Euclidean distance.
2. Visualization of the correlation coefficients and scatter plots for two representative brain systems, i.e. the VIS network regarding topological distance and the DAN regarding spatial distance.
