# Supplementary material for: Low-dimensional controllability of brain networks
Source: PLoS Comput Biol. 2025 Jan 7;21(1):e1012691. doi: 10.1371/journal.pcbi.1012691 (PMC11706394; doi:10.1371/journal.pcbi.1012691)
Supplement: S8 Fig — a) The 17 split components of the Yeo2011 brain atlas parcellation [36]. b) Adjacency matrix of the group-averaged controllability meta-graph between the different brain systems. The links represented by color are the group-average of control centrality of the system in column i when targeting system in line j λminEIGi→j. λminEIGi→j is obtained by taking the geometric mean control centrality λminEIG of drivers in system i when targeting system in j. Self-loops and the SUB network are not represented as their control centrality is several orders of magnitude higher. c) System total contribution as the sum of outgoing and incoming weighted links from the individual meta-graphs. Bars indicate group-average values and error bars standard deviations. d) System control unbalance as the difference between the sum of outgoing and incoming weighted links from the individual meta-graphs. Positive values = tendency to act as driver. Negative value = tendency to act as target. Bars indicate group-average values and error bars standard deviations. (DOCX) [file pcbi.1012691.s009.docx]

S8 Fig. Control relationships between brain systems in a finer parcellation.

1. The 17 split components of the Yeo2011 brain atlas parcellation.
2. Adjacency matrix of the group-averaged controllability meta-graph between the different brain systems. The links represented by color are the group-average of control centrality of the system in column *i* when targeting system in line *j*  $\left\langle\lambda_{min}^{EIG} \right\rangle_{i\to j}$. $\left\langle\lambda_{min}^{EIG} \right\rangle_{i\to j}$ is obtained by taking the geometric mean control centrality $\lambda_{min}^{EIG}$ of drivers in system *i* when targeting system in *j.* Self-loops and the SUB network are not represented as their control centrality is several orders of magnitude higher.
3. System total contribution as the sum of outgoing and incoming weighted links from the individual meta-graphs. Bars indicate group-average values and error bars standard deviations.
4. System control unbalance as the difference between the sum of outgoing and incoming weighted links from the individual meta-graphs. Positive values=tendency to act as driver. Negative value= tendency to act as target. Bars indicate group-average values and error bars standard deviations.
