# Supplementary material for: Low-dimensional controllability of brain networks
Source: PLoS Comput Biol. 2025 Jan 7;21(1):e1012691. doi: 10.1371/journal.pcbi.1012691 (PMC11706394; doi:10.1371/journal.pcbi.1012691)
Supplement: S9 Fig — a) Group-averaged spatial distribution of node strength k given by the sum of all the weighted links of a node. b) Group-averaged spatial distribution of low-dimensional (λminEIG) control centrality. c) Scatter plot of node strength k and low-dimensional (λminEIG) control centrality for all n × N nodes and participants. Pearson correlation test revealed a low correlation between the two metrics. (DOCX) [file pcbi.1012691.s010.docx]

|  |
| --- |
| **S9 Fig. Relationship between low-dimensional controllability and node strength.**   1. Group-averaged spatial distribution of node strength $k$ given by the sum of all the weighted links of a node. 2. Group-averaged spatial distribution of low-dimensional ($\lambda_{min}^{EIG}$) control centrality. 3. Scatter plot of node strength $k$ and low-dimensional ($\lambda_{min}^{EIG}$) control centrality for all $n\times N$ nodes and participants. Pearson correlation test revealed a low correlation between the two metrics. |
